# Supplementary material for: Whole family-based physical activity promotion intervention: the Families Reporting Every Step to Health pilot randomised controlled trial protocol
Source: BMJ Open. 2019 Oct 28;9(10):e030902. doi: 10.1136/bmjopen-2019-030902 (PMC6830702; doi:10.1136/bmjopen-2019-030902)
Supplement: Supplementary data [file bmjopen-2019-030902supp001.pdf]

Supplementary File – Parental and Young persons' questionnaires

___ ___ / ___ ___ / ___ ___ ___ ___
DD MM YYYY

TIME: 1 / 2 / 3 ID: FR __ __ __ / __ __ __ __ / __

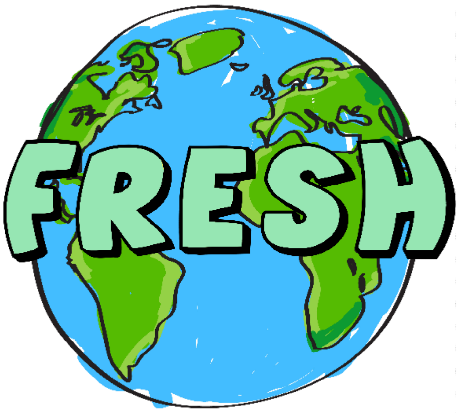


# Parent Questionnaire

- Please answer the questions as honestly and accurately as you can.
- All aspects of the study, including results, will be confidential and only the research team will have access to information on participants.
- Any reports or publications resulting from this study will not identify any individual who participated.
- Participation is entirely voluntary. There is no obligation to participate and withdrawal is permitted at any time without giving any reason and without any consequences.



Guagliano JM, *et al. BMJ Open* 2019; 9:e030902. doi: 10.1136/bmjopen-2019-030902

## Section 1: About you

**1.** In relation to the child that is between school Years 3-6 and participating in FRESH, this questionnaire is being completed by:

| | |
|---|---|
| ☐ | Mother |
| ☐ | Father |
| ☐ | Step-mother |
| ☐ | Step-father |
| ☐ | Sister |
| ☐ | Brother |
| ☐ | Grandmother |
| ☐ | Grandfather |
| ☐ | Other (e.g., guardian). Please specify: ___________________________ |

**2.** What is your sex?

| | |
|---|---|
| ☐ | Female |
| ☐ | Male |
| ☐ | Prefer not to say |

**3.** When is your date of birth?

___ ___ / ___ ___ / ___ ___ ___ ___
DD MM YYYY

**4.** What is your ethnic origin? (Please tick **ONE** box only).

| | |
|---|---|
| ☐ | White or White British |
| ☐ | Black or Black British |
| ☐ | Asian or Asian British |
| ☐ | Mixed |
| ☐ | Other ethnic group: _________________________________ |
| ☐ | Don't know |
| ☐ | Prefer not to answer |

**5.** At what age did you finish full time education? _________ years.

| | |
|---|---|
| ☐ | I have not finished full time education yet. |

**6.** What best describes your current marital status? (Please tick the box which is most applicable).

| | | | |
|---|---|---|---|
| ☐ | Single | ☐ | Separated |
| ☐ | Married or living as married | ☐ | Divorced |
| ☐ | Widowed | | |



Guagliano JM, *et al. BMJ Open* 2019; 9:e030902. doi: 10.1136/bmjopen-2019-030902

ID: FR __ __ __ / __ __ __ __ / __

**7.** Describing your health **TODAY**. Under each heading, please tick the **ONE** box that best describes your health **TODAY**.

A. **Mobility (walking about)**

| | |
|---|---|
| ☐ | I have no problems walking about today. |
| ☐ | I have slight problems walking about today. |
| ☐ | I have moderate problems walking about today. |
| ☐ | I have severe problems walking about today. |
| ☐ | I am unable to walk about today. |

**B. Self-Care**

| | |
|---|---|
| ☐ | I have no problems washing or dressing myself today. |
| ☐ | I have slight problems washing or dressing myself today. |
| ☐ | I have moderate problems washing or dressing myself today. |
| ☐ | I have severe problems washing or dressing myself today. |
| ☐ | I am unable to wash or dress myself today. |

**C. Usual activities (for example, work, study, housework, family or leisure activities).**

| | |
|---|---|
| ☐ | I have no problems doing my usual activities today. |
| ☐ | I have slight problems doing my usual activities today. |
| ☐ | I have moderate problems doing my usual activities today. |
| ☐ | I have severe problems doing my usual activities today. |
| ☐ | I am unable to do usual activities today. |

**D. Pain/discomfort**

| | |
|---|---|
| ☐ | I have no pain or discomfort today. |
| ☐ | I have slight pain or discomfort today. |
| ☐ | I have moderate pain or discomfort today. |
| ☐ | I have severe pain or discomfort today. |
| ☐ | I have extreme pain or discomfort today. |

**e. Anxiety/Depression**

| | |
|---|---|
| ☐ | I am not anxious or depressed today. |
| ☐ | I am slightly anxious or depressed today. |
| ☐ | I am moderately anxious or depressed today. |
| ☐ | I am severely anxious or depressed today. |
| ☐ | I am extremely anxious or depressed today. |



Guagliano JM, *et al. BMJ Open* 2019; 9:e030902. doi: 10.1136/bmjopen-2019-030902

**f.** We would like to know how good or bad your health is **TODAY**. This scale is numbered from 0 to 100. Mark an X on the scale to indicate how your health is **TODAY**.

100 means the **best** health you can imagine.
0 means the **worst** health you can imagine.

Now, please write the number you marked on the scale in the box below.

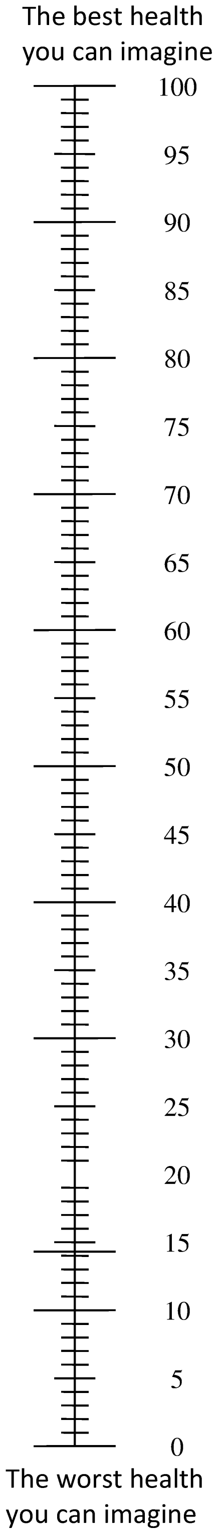


YOUR HEALTH TODAY =



Guagliano JM, *et al. BMJ Open* 2019; 9:e030902. doi: 10.1136/bmjopen-2019-030902

ID: FR __ __ __ / __ __ __ __ / __

**8.** According to national recommendations adults should be active at a **moderate intensity** (e.g., cycling or fast walking) for **at least 150 minutes every week**.

Please indicate whether you think you have achieved this level of physical activity **over the last month**:

| | |
|---|---|
| | **Yes** |
| | **No** |

**9.** Why do you engage in physical activity? Please indicate how true or untrue these statements are to you. Please circle **ONE** number on each line.

| | **Not true for me** | | **A little true for me** | | **Very true for me** |
|---|---|---|---|---|---|
| I feel guilty when I'm not physically active. | 0 | 1 | 2 | 3 | 4 |
| I value the benefits of physical activity. | 0 | 1 | 2 | 3 | 4 |
| I feel ashamed when I don't do the physical activity I planned to do. | 0 | 1 | 2 | 3 | 4 |
| It's important to me to be physically active regularly. | 0 | 1 | 2 | 3 | 4 |
| I can't see why I should bother being physically active. | 0 | 1 | 2 | 3 | 4 |
| I am physically active because others will not be pleased with me if I don't. | 0 | 1 | 2 | 3 | 4 |
| I don't see the point in being physically active. | 0 | 1 | 2 | 3 | 4 |
| I find physical activity to be enjoyable. | 0 | 1 | 2 | 3 | 4 |
| I feel under pressure from my friends/family to be physically active. | 0 | 1 | 2 | 3 | 4 |
| I feel content from participating in physical activity. | 0 | 1 | 2 | 3 | 4 |



Guagliano JM, *et al. BMJ Open* 2019; 9:e030902. doi: 10.1136/bmjopen-2019-030902

**10.** On average **over the last 4 weeks**, how much time did you spend on: (a) TV/video viewing and (b) computer use?

a. TV viewing or video watching. Please tick **ONE** box on each line.

| **Hours of TV, DVD, or video watched (not on computer/ iPad, etc.) per day** | **Average over the last 4 weeks** | | | | | |
|---|---|---|---|---|---|---|
| | **None** | **Less than 1 hour per day** | **1 to 2 hours per day** | **2 to 3 hours per day** | **3 to 4 hours per day** | **More than 4 hours per day** |
| On a weekday before 6 pm | | | | | | |
| On a weekday after 6 pm | | | | | | |
| On a weekend before 6 pm | | | | | | |
| On a weekend after 6 pm | | | | | | |

b. Computer use at home, but **not at work**. Please tick **ONE** box on each line. This includes use of: desktop computers or laptops, iPads, PlayStation, Xbox, Nintendo Wii or DS.

| **Hours of home computer use or video watched (on computer/ iPad, etc.) per day** | **Average over the last 4 weeks** | | | | | |
|---|---|---|---|---|---|---|
| | **None** | **Less than 1 hour per day** | **1 to 2 hours per day** | **2 to 3 hours per day** | **3 to 4 hours per day** | **More than 4 hours per day** |
| On a weekday before 6 pm | | | | | | |
| On a weekday after 6 pm | | | | | | |
| On a weekend before 6 pm | | | | | | |
| On a weekend after 6 pm | | | | | | |



Guagliano JM, *et al. BMJ Open* 2019; 9:e030902. doi: 10.1136/bmjopen-2019-030902

ID: FR __ __ __ / __ __ __ __ / __

## Section 2: About your child

In this section, '*your child*' is referring to your child in school Years 3-6 taking part in FRESH.

**11.** How physically active would you say **your child** is? Please tick one.

| | |
|---|---|
| | Very inactive. |
| | Fairly inactive. |
| | Neither inactive or active. |
| | Fairly active. |
| | Very active. |

**12.** Which of the following activities did **your child** do in the past 7 days? Please put '0' if not applicable.

| Did **your child** do the following activities in the past 7 days? | | **Monday-Friday**<br><br>**Total hours/minutes** | **Saturday-Sunday**<br><br>**Total hours/minutes** |
|---|---|---|---|
| **Example:**<br>Watching TV/videos | **No**<br>**Yes** (circled) | **15 hrs** | **6 hrs 30 mins** |
| Playing video games (e.g. PlayStation/Xbox/Nintendo DS). | No<br>Yes | | |
| Using computer/tablet. | No<br>Yes | | |
| Watching TV/DVD/video. | No<br>Yes | | |
| Playing games on the phone. | No<br>Yes | | |
| Other (please state):<br>__________________________. | No<br>Yes | | |

**13.** Please circle **ONE** response indicating to how much you agree or disagree with each of these statements.

| | **Strongly disagree** | | | **Strongly agree** |
|---|---|---|---|---|
| I limit how long my child plays video games (including PlayStation, Xbox, and Gameboy). | 1 | 2 | 3 | 4 |
| I limit how long my child can use the computer for things other than homework (such as playing computer games and surfing the internet). | 1 | 2 | 3 | 4 |
| I limit how long my child can watch TV or DVDs each day (including educational and non-educational programs). | 1 | 2 | 3 | 4 |



Guagliano JM, *et al. BMJ Open* 2019; 9:e030902. doi: 10.1136/bmjopen-2019-030902

## Section 3: About your family

**14.** In an **average week**, how often do you do these activities **together as a family** (i.e., joint activities including at least one child and one other family member)? Please tick **ONE** box on each line.

| | Number of Times each week | | |
|---|---|---|---|
| | **0 times each week** | **1-3 times each week** | **4 or more times each week** |
| Play active games (like playing sports or tag). | | | |
| Go to the park. | | | |
| Play board games or cards. | | | |
| Go for a bike ride. | | | |
| Go for a walk or hike. | | | |
| Watch TV or movies. | | | |
| Have family talks. | | | |
| Go swimming. | | | |
| Walk the pet(s). | | | |
| Visit family or friends. | | | |
| Eat meals together. | | | |
| Walk or bike to school. | | | |
| Play computer or video games (like PlayStation/Xbox/Nintendo DS). | | | |
| **Other** (please state)**:** ___________________________. | | | |
| **Other** (please state)**:** ___________________________. | | | |
| **Other** (please state)**:** ___________________________. | | | |

**15.** I think my family should engage in physical activity regularly (on most or all days of the week) during their free time. Please circle **ONE**.

| | |
|---|---|
| | Strongly disagree. |
| | Somewhat disagree. |
| | Neither agree or disagree. |
| | Somewhat agree. |
| | Strongly agree. |



Guagliano JM, *et al. BMJ Open* 2019; 9:e030902. doi: 10.1136/bmjopen-2019-030902

ID: FR __ __ __ / __ __ __ __ / __

**16.** Please circle **ONE** number to indicate how frequently or infrequently you do the following.

| | **Never** | **Rarely** | **Sometimes** | **Often** | **Very often** |
|---|---|---|---|---|---|
| **How often…** | | | | | |
| …do you encourage someone in your family to be physically active (e.g., bike riding walking, playing sports)? | 1 | 2 | 3 | 4 | 5 |
| …are you physically active together with someone in your family? | 1 | 2 | 3 | 4 | 5 |
| …do you take someone in your family to places where they can be physically active (e.g., the park, sports practice, swimming lessons)? | 1 | 2 | 3 | 4 | 5 |
| …do you watch or cheer for someone in your family while they are doing physical activity (e.g., sports games, gymnastics/ dance competitions)? | 1 | 2 | 3 | 4 | 5 |
| …do you ask someone in your family to be physically active with you? | 1 | 2 | 3 | 4 | 5 |
| …do you tell someone in your family that they are doing well when they are doing physical activities? | 1 | 2 | 3 | 4 | 5 |



Guagliano JM, *et al. BMJ Open* 2019; 9:e030902. doi: 10.1136/bmjopen-2019-030902

___ ___ / ___ ___ / ___ ___ ___ ___
DD MM YYYY

TIME: 1 / 2 / 3 ID: FR __ __ __ / __ __ __ __ / __

# Young Persons Questionnaire
## (15 years and under)

- Please answer the questions as honestly and accurately as you can.
- This is not a test, there is no right or wrong answer to the questions.
- If we're worried about any of the answers you have given about how you feel, we'll talk to you about it and may need to share with another adult.
- If the question asks about your parents, we mean your mum or dad or other adults who live and take care of you.



Guagliano JM, *et al. BMJ Open* 2019; 9:e030902. doi: 10.1136/bmjopen-2019-030902

**Physical Activity** is any activity that increases your breathing and makes your heart beat faster.

**Physical Activity** can be done in sports, playing with friends, or walking to school.

Some examples of physical activity are running, walking quickly, cycling, skateboarding, dancing, swimming, basketball, football, gymnastics, and playing on the playground.



Guagliano JM, *et al. BMJ Open* 2019; 9:e030902. doi: 10.1136/bmjopen-2019-030902

ID: FR __ __ __ / __ __ __ __ / __

## Part 1: About you

1. When is your birthday? (For example 1st July) ___________________________

2. How old are you? ______________________ years old.

3. Are you a boy or a girl?

| | |
|---|---|
| ☐ | Girl |
| ☐ | Boy |
| ☐ | Prefer not to say |

4. The next questions ask about how you are **TODAY**. For each question, read all the choices and tick the box ☑ that is most like you today. Only tick **ONE** box for each question.

**Example:** Today I feel quite upset so I will tick this box.

**Upset**

| | |
|---|---|
| ☐ | I don't feel upset today. |
| ☐ | I feel a little bit upset today. |
| ☐ | I feel a bit upset today. |
| ✓ | I feel quite upset today. |
| ☐ | I feel very upset today. |

Now tick the **ONE** box that is most like you today.

**A.** How **worried** are you **TODAY**?

| | |
|---|---|
| ☐ | I don't feel worried today. |
| ☐ | I feel a little bit worried today. |
| ☐ | I feel a bit worried today. |
| ☐ | I feel quite worried today. |
| ☐ | I feel very worried today. |

**B.** How **sad** are you **TODAY?**

| | |
|---|---|
| ☐ | I don't feel sad today. |
| ☐ | I feel a little bit sad today. |
| ☐ | I feel a bit sad today. |
| ☐ | I feel quite sad today. |
| ☐ | I feel very sad today. |

**C.** How much **pain** are you in **TODAY?**

| | |
|---|---|
| ☐ | I don't have any pain today. |
| ☐ | I have a little bit of pain today. |
| ☐ | I have a bit of pain today. |
| ☐ | I have quite a lot of pain today. |
| ☐ | I have a lot of pain today. |

**D.** How **tired** are you **TODAY?**



Guagliano JM, *et al. BMJ Open* 2019; 9:e030902. doi: 10.1136/bmjopen-2019-030902

- [ ] I don’t feel tired today.
- [ ] I feel a little bit tired today.
- [ ] I feel a bit tired today.
- [ ] I feel quite tired today.
- [ ] I feel very tired today.

**E.** How **annoyed** are you **TODAY?**

- [ ] I don’t feel annoyed today.
- [ ] I feel a little bit annoyed today.
- [ ] I feel a bit annoyed today.
- [ ] I feel quite annoyed today.
- [ ] I feel very annoyed today.

**F.** Did you have problems with your **school work/homework** (such as reading, writing, doing lessons) **TODAY?**

- [ ] I have no problems with my schoolwork/homework today.
- [ ] I have a few problems with my schoolwork/homework today.
- [ ] I have some problems with my schoolwork/homework today.
- [ ] I have many problems with my schoolwork/homework today.
- [ ] I can’t do my schoolwork/homework today.

**G.** Did you have problems **sleeping LAST NIGHT?**

- [ ] Last night I had no problems sleeping.
- [ ] Last night I had a few problems sleeping.
- [ ] Last night I had some problems sleeping.
- [ ] Last night I had many problems sleeping.
- [ ] Last night I couldn’t sleep at all.

**H.** Did you have problems with your **daily routine** (things like eating, having a bath/shower, getting dressed) **TODAY?**

- [ ] I have no problems with my daily routine today.
- [ ] I have a few problems with my daily routine today.
- [ ] I have some problems with my daily routine today.
- [ ] I have many problems with my daily routine today.
- [ ] I can’t do my daily routine today.

**I.** How able are you to **join in activities** (things like playing with your friends, doing sports, joining in things) **TODAY**?

- [ ] I can join in with any activities today.
- [ ] I can join in with most activities today.
- [ ] I can join in with some activities today.
- [ ] I can join in with a few activities today.
- [ ] I can join in with no activities today.



Guagliano JM, *et al. BMJ Open* 2019; 9:e030902. doi: 10.1136/bmjopen-2019-030902

ID: FR __ __ __ / __ __ __ __ / __

## Part 2: You thoughts about physical activity

**REMEMBER…**

**Physical Activity** is any activity that increases your breathing and makes your heart beat faster.

Some examples of physical activity are running, brisk walking, cycling, skateboarding, dancing, swimming, basketball, football, gymnastics, and playing on the playground.

5. Compared with other boys or girls your age, would you say that you are:

| | |
|---|---|
| | Much more active. |
| | More active. |
| | About average. |
| | Less active. |
| | Much less active. |

6. How true is each sentence for you? Please circle **ONE** number each line.

| | **Not true** | **A little bit true** | **Very true** |
|---|---|---|---|
| I am active because I enjoy being active. | 1 | 2 | 3 |
| I am active because it is important to me to be active. | 1 | 2 | 3 |
| I am active because when I don't do activity I feel bad about myself. | 1 | 2 | 3 |
| I am active because if I'm not, other people will not be happy with me. | 1 | 2 | 3 |

7. How true is each sentence for you? Please circle **ONE** number each line.

| | **Not true** | **A little bit true** | **Very true** |
|---|---|---|---|
| I can decide which activities I want to do. | 1 | 2 | 3 |
| I feel I am active because I want to be. | 1 | 2 | 3 |
| When it comes to being active, I think I am pretty good. | 1 | 2 | 3 |
| I am happy with how good I am at being active. | 1 | 2 | 3 |
| When it comes to being active, I feel like I'm part of a group. | 1 | 2 | 3 |
| When it comes to being active, I fit in well with others. | 1 | 2 | 3 |



Guagliano JM, *et al. BMJ Open* 2019; 9:e030902. doi: 10.1136/bmjopen-2019-030902

## Part 3: About your parents/family

8. How often do **your parents** let you do the following? Please circle **ONE** number each line.

| | Never | Sometimes | Often |
|---|---|---|---|
| My parents let me watch TV as much as I want. | 1 | 2 | 3 |
| My parents let me use the computer as much as I want for things that aren't homework like playing games or watching videos. | 1 | 2 | 3 |
| My parents let me play video games (such as PlayStation, Xbox, and Gameboy) as much as I want. | 1 | 2 | 3 |

9. How often does **your family** do the following? Please circle **ONE** number each line.

| | Never | Sometimes | Often |
|---|---|---|---|
| How often does someone in your family encourage (or tell) you to do active things (like bike riding walking, playing sports)? | 1 | 2 | 3 |
| How often are you active together with someone in your family? | 1 | 2 | 3 |
| How often does someone in your family bring you to places where you can be active (like the park, sports practice, swimming lessons)? | 1 | 2 | 3 |
| How often does someone in your family watch or cheer for you when you are doing active things (like sports games or gymnastics or dance competitions)? | 1 | 2 | 3 |
| How often does someone in your family ask you to be active with them? | 1 | 2 | 3 |
| How often does someone in your family tell you that you are doing good when you are doing active things? | 1 | 2 | 3 |



Guagliano JM, *et al. BMJ Open* 2019; 9:e030902. doi: 10.1136/bmjopen-2019-030902

ID: FR __ __ __ / __ __ __ __ / __

10. In a normal week, how many times do you do these activities together as a family? Please tick **ONE** box every line.

| | **Number of Times each week** | | |
|---|---|---|---|
| | **0 times each week** | **1-3 times each week** | **4 or more times each week** |
| Play active games (like playing sports or tag). | | | |
| Go to the park. | | | |
| Play board games or cards. | | | |
| Go for a bike ride. | | | |
| Go for a walk or hike. | | | |
| Watch TV or movies. | | | |
| Have family talks. | | | |
| Go swimming. | | | |
| Walk the pet(s). | | | |
| Visit family or friends. | | | |
| Eat meals together. | | | |
| Walk or bike to school. | | | |
| Play computer or video games (like PlayStation/Xbox/Nintendo DS). | | | |
| **Other:** ___________________________. | | | |
| **Other:** ___________________________. | | | |

11. How true is each sentence? Please fill in **ONE** circle in each line.

| | **Not true** | **A little bit true** | **Very true** | **Does not apply** |
|---|---|---|---|---|
| My Mum thinks that I should be active during my free time on most days of the week. | 1 | 2 | 3 | |
| My Dad thinks that I should be active during my free time on most days of the week. | 1 | 2 | 3 | |
| My sister(s) think that I should be active during my free time on most days of the week. | 1 | 2 | 3 | |
| My brother(s) think that I should be active during my free time on most days of the week. | 1 | 2 | 3 | |



Guagliano JM, *et al. BMJ Open* 2019; 9:e030902. doi: 10.1136/bmjopen-2019-030902
