# Supplementary material for: Whole family-based physical activity promotion intervention: the Families Reporting Every Step to Health pilot randomised controlled trial protocol
Source: BMJ Open. 2019 Oct 28;9(10):e030902. doi: 10.1136/bmjopen-2019-030902 (PMC6830702; doi:10.1136/bmjopen-2019-030902)
Supplement: Author's manuscript [file bmjopen-2019-030902.draft_revisions.pdf]

BMJ Open is committed to open peer review. As part of this commitment we make the peer review history of every article we publish publicly available.

When an article is published we post the peer reviewers' comments and the authors' responses online. We also post the versions of the paper that were used during peer review. These are the versions that the peer review comments apply to.

The versions of the paper that follow are the versions that were submitted during the peer review process. They are not the versions of record or the final published versions. They should not be cited or distributed as the published version of this manuscript.

BMJ Open is an open access journal and the full, final, typeset and author-corrected version of record of the manuscript is available on our site with no access controls, subscription charges or pay-per-view fees (http://bmjopen.bmj.com).

If you have any questions on BMJ Open's open peer review process please email info.bmjopen@bmj.com

BMJ Open

A whole family-based physical activity promotion intervention: The Families Reporting Every Step to Health (FRESH) pilot randomised controlled trial protocol

Journal:	*BMJ Open*
Manuscript ID	bmjopen-2019-030902
Article Type:	Protocol
Date Submitted by the Author:	08-Apr-2019

Complete List of Authors:	Guagliano, Justin; University of Cambridge, MRC Epidemiology Unit and Centre for Diet and Activity Research<br>Brown, Helen; University of Cambridge, MRC Epidemiology Unit and Centre for Diet and Activity Research<br>Coombes, Emma; University of East Anglia, Norwich Medical School and UKCRC Centre for Diet and Activity Research<br>Haines, Elizabeth; University of Cambridge, MRC Epidemiology Unit and Centre for Diet and Activity Research<br>Hughes, Claire ; University of Cambridge, Centre for Family Research<br>Jones, Andrew; University of East Anglia, Norwich Medical School and UKCRC Centre for Diet and Activity Research<br>Morton, Katie; University of Cambridge, MRC Epidemiology Unit and UKCRC Centre for Diet and Activity Research<br>van Sluijs, Esther; University of Cambridge, MRC Epidemiology Unit and UKCRC Centre for Diet and Activity Research

Keywords:	co-participation, co-physical activity, youth, parents

SCHOLARONE™
Manuscripts

A whole family-based physical activity promotion intervention: The Families Reporting Every Step to Health (FRESH) pilot randomised controlled trial protocol

Justin M. Guagliano[1§], Helen Elizabeth Brown[1], Emma Coombes[2], Elizabeth S. Haines[1], Claire Hughes[3], Andy P. Jones[2], Katie L. Morton[1], & Esther M. F. van Sluijs[1]

[1]MRC Epidemiology Unit and UKCRC Centre for Diet and Activity Research, University of Cambridge, Cambridge, UK

[2]Norwich Medical School and UKCRC Centre for Diet and Activity Research, University of East Anglia, Norwich, UK

[3]Centre for Family Research, University of Cambridge, Cambridge, UK

[§]Corresponding author

Author email addresses:

JMG: Justin.Guagliano@mrc-epid.cam.ac.uk

HEB: Helen.Brown@bi.team

EC: Emma.Coombes@uea.ac.uk

ESH: Beth.Haines@mrc-epid.cam.ac.uk

CH: ch288@cam.ac.uk

APJ: A.P.Jones@uea.ac.uk

KLM: Katie.Morton@innoviatech.com

EvS: Esther.vanSluijs@mrc-epid.cam.ac.uk

For peer review only - http://bmjopen.bmj.com/site/about/guidelines.xhtml

Abstract

Introduction: Family-based physical activity (PA) interventions may present a promising avenue to promote children's activity, however, high-quality experimental research is lacking. This paper describes the protocol for the FRESH (Families Reporting Every Step to Health) pilot trial, a child-led family-based PA intervention delivered online.

Methods and analysis: FRESH is an 8-week three-armed, parallel-group, randomised controlled pilot trial, using a 1:1:1 allocation ratio and long-term follow-up (52 weeks post-baseline). Families will be eligible if a minimum of one child in school Years 3-6 (aged 7-11 years) and at least one adult responsible for that child are willing to participate. Family members can take part in the intervention irrespective of their participation in the accompanying evaluation and vice versa.

Following baseline assessment, families will be randomly allocated to one of three arms: (1) FRESH, (2) pedometer-only, or (3) no-intervention control. All family members in the pedometer-only and FRESH arms receive pedometers and generic family PA promotion information. FRESH families additionally receive access to the intervention website; allowing participants to select step challenges to 'travel' to target cities around the world, log steps, and track progress as they virtually globetrot. Control families will receive no treatment. All family members will be eligible to participate in the evaluation with two follow-ups (8 and 52 weeks). Physical (e.g., fitness, blood pressure), psychosocial (e.g., social support), and behavioural (e.g., objectively-measured family PA) measures will be collected each time point. At 8-week follow up, a mixed-methods process evaluation will be conducted (questionnaires and family focus groups) assessing acceptability of the intervention. FRESH families' website engagement will also be explored.

Ethics and dissemination: This study received ethical approval from the Ethics Committee for the School of the Humanities and Social Sciences at the University of Cambridge. Findings will be disseminated via peer-reviewed publications, conferences, and to participating families.

Trial registration number: This study was prospectively registered (ISRCTN12789422). Registered 16 March 2016. http://www.isrctn.com/ISRCTN12789422.

Keywords: youth, parent, mothers, fathers, mums, dads, co-participation, co-physical activity

For peer review only

Article Summary

Strengths and limitations of this study

- This trial is among the first physical activity interventions that specifically targets and evaluates whole family engagement.
- Novel methodological work will be completed to compute family co-participation in physical activity using objective measures.
- The use of mixed-methods will provide unique insight and context for our quantitative findings.
- A long-term follow up (52 weeks post-baseline) will enable assessment of the potential for long-term effects and an accurate assessment of long-term participant retention, which is notoriously challenging in child-based research.
- However, this pilot trial will not allow us to draw conclusions of effectiveness.

For peer review only

For peer review only - http://bmjopen.bmj.com/site/about/guidelines.xhtml

Introduction

Although the benefits of regular physical activity in children are well-established [1, 2], roughly only half of UK children meet the recommended 60 minutes of daily moderate-to-vigorous intensity physical activity (MVPA) [3]. Moreover, observational data reveal that children are less active after school and at weekends compared to during school time [4-6], and that MVPA declines steeply as children enter adolescence [7], particularly at weekends [6].

Targeting families may therefore be a valuable way to increase physical activity among children [8, 9]. In particular, parents can influence their children's health behaviours through a variety of mechanisms, including their general parenting style, parenting practices (e.g., rule setting, behavioural consequences, establishing behavioural expectations), and their control over the home environment [10, 11]. Parents can also act as gatekeepers to activity [12] and can play an important role in increasing their child's physical activity through role-modelling or parental support [13]. However, little physical activity promotion research has been conducted in the family compared to the school setting [14, 15]. Previous research suggests that: (1) involving family members is critical for sustained behaviour change [13, 16, 17]; and (2) home-based physical activity interventions that include the family are potentially more effective than those requiring the family to travel to community or other intervention locations [18, 19]. Many studies, however, only focus on promoting child physical activity instead of considering the family as a unit that may work together to change behaviour [20].

Our recent feasibility study [21] was among the first physical activity interventions to specifically target whole family engagement. The findings showed that it was feasible and acceptable to deliver and evaluate a family-targeted physical activity promotion intervention with high acceptability from participating families. Building upon this work, this paper describes the protocol for the Families Reporting Every Step to Health (FRESH) pilot trial. The purpose of this three-armed randomised controlled pilot study is to assess: the feasibility and acceptability of the revised recruitment strategy, long-term retention, FRESH intervention and outcome evaluation (after feasibility testing [21]) and to explore further intervention optimisation and preliminary effectiveness.

For peer review only - http://bmjopen.bmj.com/site/about/guidelines.xhtml

Methods and Analysis

The Standard Protocol Items: Recommendations for Interventional Trials [22] is used to guide the reporting of this study. We also use the Template for Intervention Description and Replication [23] to guide our description of the intervention.

Trial design

FRESH is an 8-week three-armed, parallel-group, randomised controlled pilot trial, using a 1:1:1 allocation ratio and long-term follow-up (52 weeks post-baseline; see Figure 1). Following baseline assessment, families will be randomly allocated to one of three arms: (1) FRESH arm, (2) pedometer-only arm, or (3) no-intervention control arm. All family members in the pedometer-only and FRESH arms receive pedometers and generic family physical activity promotion information (described later). FRESH families additionally receive access to the intervention website (described later). Control families will be asked to carry on as normal.

Participants

Families are deemed eligible to participate in this study if there is consent from at least one child in school Years 3-6 (aged 7-11 years, hereafter referred to as the index child) and at least one adult responsible for the index child. The adult must live in the main household with the index child. There are no restrictions placed on family type (e.g., single parent, inclusion of extended family living in the main household) and no maximum number of participants per family. All participants need to be able to perform light-intensity physical activity (e.g., walking), have access to the Internet, and understand the English language sufficiently well to provide informed consent. Family members are able to take part in the intervention irrespective of their participation in the accompanying evaluation and vice versa. Exclusion criteria related to participation in specific assessments are outlined below.

Setting

Families will be recruited from rural Norfolk and Suffolk, counties in East Anglia, United Kingdom. Norfolk is 2,074 square miles in size and had a total estimated population of 898,400 in

For peer review only - http://bmjopen.bmj.com/site/about/guidelines.xhtml

2017 (most recent estimate) [24] and Suffolk is 1,466 square miles and had a total estimated population of 756,978 in 2017 (most recent estimate) [25]. According to the Norfolk and Suffolk County Councils [26, 27], approximately 53% and 42% of the Norfolk and Suffolk populations respectively are classified as living in a rural area. Based on the Office for National Statistics [28] classification, 'rural' will be defined as having a postcode falling in a small town, village, hamlet, or dispersed settlement. In both counties, existing inequalities have been identified, including physical activity, obesity and other indicators of child ill health, school readiness, and attainment [6, 29, 30].

Recruitment

The recruitment of families is known to be particularly challenging [11, 13] and we have described specific recruitment challenges we have encountered previously [21]. To overcome these challenges, we will use a multi-faceted recruitment strategy, including targeting adults (parents) directly, and messaging that focuses on the wider benefits of research participation (e.g., spending more time together as a family) as opposed to solely focusing on increasing physical activity or obesity prevention [31]. Recruitment will be undertaken using two main strategies that target adults and children, as summarised in Figure 2. The first strategy involves recruitment in the school setting and the second in employer-based and community-based settings (e.g., Brownies/Cubs, community centres, GP surgeries). Alternative recruitment settings may also be explored (e.g., online/traditional media) if needed, and will follow the same procedure as the second strategy. For logistical purposes, we aim to find recruitment settings located roughly within an hour commute of Cambridge, United Kingdom.

Recruitment protocol. To recruit schools, employers, and community-based organisations, we will aim to first contact those in lead positions (e.g., head teachers, human resources, health and wellbeing leaders, heads of community-based organisations). An information pack detailing the purpose of the study and all procedures will be included in our correspondence with lead personnel. Also included in the information pack and recruitment material will be a link to a recruitment video, which was developed with families following the suggestion of participants in the FRESH feasibility study [21]. For school-based recruitment, verbal or written approval will be sought to send home

For peer review only - http://bmjopen.bmj.com/site/about/guidelines.xhtml

study leaflets with children, circulate our leaflet to parents online, and send an online reminder to parents approximately two weeks later. We will also seek permission to present to Key Stage 2 students (Years 3-6; 7-11 years) at a scheduled school assembly. Similarly, for employer- and community-based recruitment, we will seek approval to circulate our study information to employees or members either online or hardcopy. In all cases, interested parents are asked to express interest by contacting the study team via e-mail or a free-to-call telephone number, after which eligibility will be assessed and study information emailed. A baseline assessment appointment will be made with families still interested in participating. Written informed consent will be obtained for all participating adults and written parental consent and child assent for each participating child during this appointment, prior to baseline assessments.

Retention. To encourage retention, we will remain in regular contact with all participating families (through intervention website, newsletter/holiday cards), and offer measurement incentives and study feedback. Each participant will receive a £5 voucher upon return of the accelerometer and GPS monitors at each measurement time point. Retention will be monitored by study group and demographic characteristics to observe whether differences in retention occur.

Randomisation

Randomisation will occur after baseline assessments and the unit of randomisation will be at the family level (i.e., the index child and all participating family members). Families will be randomised in blocks of six by an independent statistician using a computer generated algorithm produced with Stata (version 14; StataCorp. TX: StataCorp LP) and stratified by county (i.e., Norfolk or Suffolk). The random allocation sequence will be implemented via a database created in-house on Microsoft Access by independent data management staff. A study coordinator will use the database after baseline to determine which study arm a family is allocated to. No one directly involved in the evaluation will have access to the allocation code or complete sequence.

Intervention protocol for FRESH arm families

For peer review only - http://bmjopen.bmj.com/site/about/guidelines.xhtml

The development, feasibility, acceptability, and refinements made to the intervention prior to the current pilot trial have been previously described [21]. In brief, the intervention the FRESH arm will receive is primarily a goal-setting and self-monitoring intervention, delivered online, aimed at increasing physical activity in whole families. The intervention uses concepts from the Socio-Ecological Model [32] and Family Systems Theory [33] and operationalises constructs from Self-Determination Theory [34] to inform behaviour change strategies. A detailed description of the FRESH intervention components, behaviour change techniques, targeted theoretical constructs, and hypothesised mediators are in Table 1. Additionally, the FRESH logic model can be found in Figure 3.

A week after baseline measures, families allocated to the FRESH arm will have an hour-long kick-off meeting, scheduled in the family home with a facilitator. The facilitator will introduce families to the intervention components, accompanying materials (e.g., family action planner), and distribute pedometers. Families will also receive their first of four pieces of generic walking information (described in a subsequent section). However, the main goals of this meeting are to familiarise families with the intervention website and their pedometers and to prompt weekly 'family time' meetings (described in Table 1) in which they complete their action planners and select a new challenge city to 'walk to' on the FRESH intervention website. All families will receive a follow up phone call a week after their kick-off meeting to discuss any issues or ask any clarifying questions. Participant-initiated distant support will be available for the duration of the intervention, where participants can contact the research team with questions or to report issues (e.g., website bugs, pedometer issues).

Table 1 provides a detailed description of the FRESH intervention components. In each family, the index child will be designated the role of 'team captain', which involves taking the lead in selecting challenges and uploading steps online. Evidence suggests that children may act as change agents to elicit changes to the psychosocial environment [14]; therefore, promoting the index children to the role of family 'team captain' may strengthen child buy-in, perceived autonomy, and improve intervention fidelity. All family members will be given pedometers, wearing them for as long as possible daily, to capture their steps during challenges. Pedometers are simple to use, convenient, and

For peer review only - http://bmjopen.bmj.com/site/about/guidelines.xhtml

For peer review only

are associated with effective interventions for increasing parent-child physical activity [e.g., 35]. After the challenge week is over, whether a family completes their challenge or not, they will receive personalised competence reinforcement messages praising their effort online and hardcopy letters (addressed and mailed to all participating children in the family). In addition, online and tangible rewards will be given to participating children after a challenge week. If a family does not complete a challenge, they will progress to a hidden city along their challenge route, as opposed to the city they chose, and will still receive a reinforcement message and reward, as described above. Families with an ongoing challenge will receive email reminders to log steps three days and one day before an impending challenge ends. After every challenge week, the above cycle will repeat starting with the next 'family time' meeting. In this way, it will take families a minimum of 12 weeks to travel around the world. Following T2 (8 weeks post-baseline), families retain access to the website and pedometers, and can continue using it for as long as they like.

Protocol for pedometer-only and control families during the 8-week intervention period

Following the baseline assessment, families allocated to the pedometer-only arm will be mailed pedometers and generic family physical activity promotion information produced by Walk4Life, a sub-brand of Change4Life (www.nhs.uk/change4life). Information will continue to be emailed to families (pedometer-only and FRESH arm families) fortnightly for the duration of the 8-week intervention period. The information will provide families with tips to get walking daily and games that can be played while walking. Control families will be asked to carry on as normal and will not receive access to the intervention website, pedometers, or any generic information.

Outcome evaluation measures

Table 2 outlines the measures taken, including assessment order and estimated duration. Data collection will be carried out by two trained research staff and will occur in participating families' homes. Outcomes will be assessed at baseline (prior to randomisation), 8 weeks post-baseline, and follow up (52 weeks post-baseline) on all consenting family members (excluding children ≤ 2 years).

Primary outcome

For peer review only - http://bmjopen.bmj.com/site/about/guidelines.xhtml

Physical activity outcomes. To assess physical activity, participants will be asked to simultaneously wear an ActiGraph GT3X+ tri-axial accelerometer (ActiGraph LLC; Pensacola, Florida) and QStarz Travel Recorder BT1000X global positioning system (GPS) monitor (QStarz; Taipei, Taiwan). The accelerometer will be initialised to record data at a sampling rate of 50 Hz, while the GPS will be set to record a location every 10 seconds. The devices will be worn on each hip during waking hours for 7 consecutive days. A valid week will be defined as a minimum of 420 minutes/day from 3 days over the 7-day measurement period; however, this definition may be altered depending on participant compliance. Non-wear will be defined as ≥ 90 mins consecutive zeros using the vector magnitude. Raw accelerometer counts will be downloaded and integrated into 5-second epochs. Evenson et al. cut-points [36] have been recommended to estimate physical activity intensity in youth [37, 38] and Troiano et al. [39] cut-points for adults, these cut-points will be used in this study. In addition, family co-participation in physical activity will be measured by matching accelerometer and GPS data using Java. Novel methodological work will be completed to compute minutes of physical activity per day when family members are together at a given location from the matched data.

Secondary outcomes

Health outcomes. Aerobic fitness will be measured using an 8-minute submaximal step test (with 2-minute rest), which provides an individual calibration of heart rate to work rate (energy expenditure per unit time) to predict a fitness estimate of a participants' heart rate recovery index [40]. Children < 7 years will be excluded from the aerobic fitness test.

Height and weight will be measured once with a Leicester portable stadiometer and Seca 877 digital scale, respectively. Waist circumference will be measured twice, using a non-elastic tape measure. A third measure is taken if the first two measures differed by ≥ 3 cm. Body mass index will be calculated, and converted into age- and sex-specific percentiles using standard growth charts for children [41].

Behavioural and psychosocial measures. Behavioural and psychosocial measures will be measured via questionnaire distributed to adults and children (those ≤ 4 years will not complete questionnaires). These will include: adult and child screen-time use [42-45]; quality of life [46-49];

For peer review only - http://bmjopen.bmj.com/site/about/guidelines.xhtml

family co-participation in physical activity [45]; physical activity awareness [50, 51]; family social norms for physical activity [52, 53]; family support [52]; children's and adult's motivation for physical activity [54, 55]; and children's perceived autonomy, competence, and relatedness [55].

Family functioning. The Fictional Family Holiday Paradigm will be used to assess family functioning via family relationships [56] and connectedness [57]. In this observational paradigm, each family is asked to spend 10 minutes planning and discussing a week-long holiday itinerary with unlimited budget. The video-recorded activity is then transcribed prior to coding family functioning, which is indexed through multiple conversational markers, including: 'power sharing' (i.e., taking turns); parental elicitation of all family members' viewpoints; expressions of individuality (e.g., suggestions for destinations/activities or disagreements) and connectedness (e.g., agreements, questions, or initiating compromise) [57]; as well as non-verbal markers, including expressions of amity (e.g., laughter, displays of affection) or hostility (e.g., sarcasm, anger).

Family out-of-pocket expenditure for physical activity. Physical activity related expenditure for each family member will be collected via a questionnaire that was developed and refined following our feasibility study [21]. The questionnaire comprises two questions about expenditure related to membership fees and subscriptions (e.g., for sports clubs, fitness centres) and sports equipment (e.g., sportswear, gadgets) and is completed by the same adult at each time point for their whole family.

Process evaluation

A mixed-methods process evaluation will be conducted after T2 (8-weeks post-baseline). Using opened-ended and 4-point Likert-scale questions (1 = strongly disagree, 4 = strongly agree), adults will self-report their overall opinion of FRESH, the intervention components, measurements, and suggestions for improvement. Children will also self-report on the above topics, responding to dichotomous 'yes/no' questions. In addition, semi-structured focus groups will be conducted online on 10/20 FRESH families and 5/20 families from each of the other two arms (i.e., pedometer-only, control). Focus groups will focus on families' experience taking part in the trial, perceived acceptability of individual intervention components, intervention fidelity, challenges/barriers

For peer review only

For peer review only - http://bmjopen.bmj.com/site/about/guidelines.xhtml

encountered, and suggested improvements, as appropriate based on study arm allocation. All focus groups will be transcribed verbatim. We will also explore FRESH families' engagement with the intervention website (e.g., page views, challenges accepted/completed).

Patient and public involvement

FRESH was developed with substantial input from children and families from the public, which has been described elsewhere [21]. Since the completion of the FRESH feasibility study, families from the public have been further involved with the optimisation of FRESH in a number of ways. As mentioned above, we sought the involvement of families from the public to develop a recruitment video; these families helped develop the script and acted in the video, which can be viewed here: www.youtube.com/watch?v=UxUHN1JsjUM. We also asked families to engage with the FRESH website and provide feedback to inform modifications that could be made. Lastly, we have included parents as members on our Study Steering Committee.

Blinding

Researchers conducting analyses will be blinded to family treatment allocation. Research assistants will not be explicitly told family treatment allocation. However, it will not be possible to conceal treatment allocation from participating families.

Sample size considerations

Since this is a pilot study, a sample size calculation will not be performed. We plan to recruit a sample of 60 families, with a sample size of ~180-240 participants based ~3-4 members per family. Our estimated sample size is based on prior study experience [31] and sample sizes of previous pilot studies [58, 59].

Data analysis

Quantitative data. Statistical analyses of the primary and applicable secondary outcomes will be conducted using linear mixed models in Stata (version 14; StataCorp. TX: StataCorp LP). The

For peer review only - http://bmjopen.bmj.com/site/about/guidelines.xhtml

models will be used to assess the impact of treatment (FRESH, pedometer-only, or control), time, (baseline, 8-weeks, and 52-weeks), and the group-by-time interaction. An estimate of effect and 95% confidence interval will be calculated. Economic analyses will include calculations of total cost per family (i.e., the sum of intervention delivery and physical activity expenses in each arm), cost-effectiveness (i.e., the incremental cost per family at each time point combined with change in MVPA), and a value of information analysis [60]. Descriptive statistics will also be calculated to describe data related to recruitment, retention, acceptability, family functioning, and website engagement.

Qualitative data. A content analysis will be conducted using existing guidelines [61] to explore the feasibility and acceptability of the revised FRESH intervention, outcome evaluation, and suggestions for further intervention optimisation via family focus groups. Specifically, the analysis will be conducted in two separate phases. During the data organisation phase, text from each transcript will be divided into segments (meaning units) to produce a set of concepts that reflected meaningful pieces of information [61]. Tags will then be assigned to each meaning unit. Tagging will be performed by one researcher, with a second double-tagging ~25% of transcripts. For the data interpretation phase, the inventory of tags from all transcripts will examined by two researchers, which will lead to the emergence of themes and sub-themes within each overarching category.

Discussion

The FRESH study will provide a response to calls for the need for innovative interventions targeting young people and families [62] and builds upon our previous work [21]. As far as we are aware, FRESH is among the first physical activity interventions that specifically targets and evaluates whole family engagement.

The overall aim of a future definitive trial will be to establish the long-term effectiveness and cost-effectiveness of the family-based FRESH intervention to promote MVPA in young people and their families. A decision to progress to a definitive trial will be made with an independent Study Steering Committee, using the following parameters and taking into account qualitative findings on the acceptability of trial procedures:

For peer review only - http://bmjopen.bmj.com/site/about/guidelines.xhtml

- Demonstrable feasibility of recruiting 20 families/month (accounting for increased staffing in future definitive trial) and retaining 75% of index children at follow-up (52 weeks post-baseline);
- Good intervention adherence for families in the FRESH arm, defined as >75% of families upload steps at least 6 times in the first 3 months of the study;
- Intervention optimisation is feasible (i.e., identified adaptations are practical, affordable, acceptable);
- Evidence to suggest an adequately powered trial would require a feasible number of participants;
- Discontinuation of trial arm based on evidence of harm or limited acceptability/feasibility;
- Positive expected net gain of sampling from definitive trial.

Ethics and dissemination

Written informed consent will be obtained for all participating adults and written parental consent and child assent for each participating child prior to collecting baseline assessments. Ethical approval has been obtained from the Ethics Committee for the School of the Humanities and Social Sciences at the University of Cambridge and this study has been prospectively registered (ISRCTN12789422).

For peer review only

List of abbreviations

FRESH, Families Reporting Every Step to Health; GPS, global positioning system; MVPA, moderate-to-vigorous physical activity.

Declarations

Authors' contributions. EvS (principal investigator), HEB, CH, APJ, and KLM secured funding for the research. All authors contributed to the study design. JMG drafted the manuscript. HEB, EC, ESH, CH, APJ, KLM, and EvS critically reviewed and revised the manuscript. All authors read and approved the final manuscript.

Funding. This work was supported by the National Institute for Health Research Public Health Research Programme (project number 15/01/19). Intervention costs for the current study were supported by Active Norfolk and Suffolk County Council. Funding was also received from the Medical Research Council (project number MC_UU_12015/7).

Competing interests. The authors declare that they have no competing interest. The views expressed are those of the author(s) and not necessarily those of the National Health Service, the National Institute for Health Research, or the Department of Health and Social Care.

Acknowledgements. This work was undertaken by the Centre for Diet and Activity Research (CEDAR), where funding from Cancer Research UK, the British Heart Foundation, the Economic and Social Research Council, the Medical Research Council, the National Institute for Health Research, and the Wellcome Trust, under the auspices of the UK Clinical Research Collaboration, is gratefully acknowledged [087636/Z/08/Z; ES/G007462/1; MR/K023187/1].

For peer review only - http://bmjopen.bmj.com/site/about/guidelines.xhtml

Figure legend.

Figure 1. Participant flow diagram.

Figure 2. Recruitment strategy.

Figure 3. FRESH theoretical model.

For peer review only

For peer review only - http://bmjopen.bmj.com/site/about/guidelines.xhtml

References

1. Janssen I, LeBlanc AG: **Systematic review of the health benefits of physical activity and fitness in school-aged children and youth**. *Int J Behav Nutr Phy.* 2010, **7**(40):1-16.

2. Poitras VJ, Gray CE, Borghese MM, Carson V, Chaput J-P, Janssen I, Katzmarzyk PT, Pate RR, Connor Gorber S, Kho ME: **Systematic review of the relationships between objectively measured physical activity and health indicators in school-aged children and youth**. *Appl Physiol Nutr Me.* 2016, **41**(6):S197-S239.

3. Griffiths LJ, Cortina-Borja M, Sera F, Pouliou T, Geraci M, Rich C, Cole TJ, Law C, Joshi H, Ness AR: **How active are our children? Findings from the Millennium Cohort Study**. *BMJ Open.* 2013, **3**(8):e002893.

4. Brooke HL, Atkin AJ, Corder K, Ekelund U, van Sluijs EM: **Changes in time-segment specific physical activity between ages 10 and 14 years: A longitudinal observational study**. *J Sci Med Sport.* 2016, **19**(1):29-34.

5. Corder K, Craggs C, Jones AP, Ekelund U, Griffin SJ, van Sluijs EM: **Predictors of change differ for moderate and vigorous intensity physical activity and for weekdays and weekends: A longitudinal analysis**. *Int J Behav Nutr Phy.* 2013, **10**:69.

6. Corder K, Sharp S, Atkin AJ, Griffin S, Jones A, Ekelund U, van Sluijs E: **Change in objectively measured physical activity during the transition to adolescence**. *Brit J Sport Med.* 2013, **49**(11):730-736.

7. Hallal PC, Andersen LB, Bull FC, Guthold R, Haskell W, Ekelund U: **Global physical activity levels: surveillance progress, pitfalls, and prospects**. *Lancet.* 2012, **380**(9838):247-257.

8. Kaushal N, Rhodes RE: **The home physical environment and its relationship with physical activity and sedentary behavior: A systematic review**. *Prev Med.* 2014, **67**:221-237.

9. Maitland C, Stratton G, Foster S, Braham R, Rosenberg M: **A place for play? The influence of the home physical environment on children's physical activity and sedentary behaviour**. *Int J Behav Nutr Phy.* 2013, **10**(1):99.

For peer review only

For peer review only - http://bmjopen.bmj.com/site/about/guidelines.xhtml

10. Ventura AK, Birch LL: **Does parenting affect children's eating and weight status?** *Int J Behav Nutr Phy.* 2008, **5**(15).

11. Morgan PJ, Jones RA, Collins CE, Hesketh KD, Young MD, Burrows TL, Magarey AM, Brown HL, Hinkley T, Perry RA: **Practicalities and research considerations for conducting childhood obesity prevention interventions with families**. *Children.* 2016, **3**(4):24-40.

12. Patrick H, Hennessy E, McSpadden K, Oh A: **Parenting styles and practices in children's obesogenic behaviors: scientific gaps and future research directions**. *Child Obes.* 2013, **9**(S1):S73-S86.

13. O'Connor TM, Jago R, Baranowski T: **Engaging parents to increase youth physical activity: A systematic review**. *Am J Prev Med.* 2009, **37**(2):141-149.

14. Brown HE, Atkin AJ, Panter J, Wong G, Chinapaw MJ, van Sluijs E: **Family-based interventions to increase physical activity in children: A systematic review, meta-analysis and realist synthesis**. *Obes Rev.* 2016, **17**(4):345-360.

15. Marsh S, Foley L, Wilks D, Maddison R: **Family-based interventions for reducing sedentary time in youth: A systematic review of randomized controlled trials**. *Obes Rev.* 2014, **15**(2):117-133.

16. van Sluijs EM, McMinn A: **Preventing obesity in primary schoolchildren**. *BMJ.* 2010, **340**:819.

17. Kipping RR, Howe LD, Jago R, Campbell R, Wells S, Chittleborough CR, Mytton J, Noble SM, Peters TJ, Lawlor DA: **Effect of intervention aimed at increasing physical activity, reducing sedentary behaviour, and increasing fruit and vegetable consumption in children: Active for Life Year 5 (AFLY5) school based cluster randomised controlled trial**. *BMJ.* 2014, **348**:348-361.

18. van Sluijs EM, Kriemler S, McMinn AM: **The effect of community and family interventions on young people's physical activity levels: A review of reviews and updated systematic review**. *Brit J Sport Med.* 2011, **45**(11):914-922.

For peer review only

For peer review only - http://bmjopen.bmj.com/site/about/guidelines.xhtml

19. Rhodes RE, Lim C: **Promoting parent and child physical activity together: elicitation of potential intervention targets and preferences**. *Health Educ Behav.* 2018, **45**(1):112-123.

20. Shonkoff JP, Fisher PA: **Rethinking evidence-based practice and two-generation programs to create the future of early childhood policy**. *Dev Psychopathol.* 2013, **25**(4 pt 2):1635-1653.

21. Guagliano JM, Brown HE, Coombes E, Hughes C, Jones AP, Morton KL, Wilson EC, van Sluijs EM: **The development and feasibility of a randomised family-based physical activity promotion intervention: the Families Reporting Every Step to Health (FRESH) study**. *Pilot and Feasibility Studies.* 2019, **5**:21.

22. Chan A-W, Tetzlaff JM, Altman DG, Laupacis A, Gøtzsche PC, Krleža-Jerić K, Hróbjartsson A, Mann H, Dickersin K, Berlin JA: **SPIRIT 2013 statement: Defining standard protocol items for clinical trials**. *Ann Intern Med* 2013, **158**(3):200-207.

23. Hoffmann TC, Glasziou PP, Boutron I, Milne R, Perera R, Moher D, Altman DG, Barbour V, Macdonald H, Johnston M: **Better reporting of interventions: template for intervention description and replication (TIDieR) checklist and guide**. *BMJ.* 2014, **348**:g1687.

24. Norfolk County Council. **Data and information about Norfolk's population.** 2015. http://www.norfolkinsight.org.uk. Accessed 8 Jan 2019.

25. Suffolk Observatory. **Population estimates.** 2018. https://www.suffolkobservatory.info/population/report/view/0c18eadb6fb544c1bee07bc7561 8ec49/E10000029/. Accessed 8 Jan 2019.

26. Norfolk County Council. **Norfolk Rural Development Strategy 2013-2020.** 2013. https://www.norfolk.gov.uk/-/media/norfolk/downloads/what-we-doand-how-we-work/policy-performance-and-partnerships/policies-andstrategies/business/norfolk-rural-development-strategy-2013-20.pdf. Accessed 8 Jan 2019.

27. Suffolk County Council. **Suffolk Demographic Profile**. 2015. https://www.suffolk.gov.uk/assets/council-and-democracy/our-aims-and-transformation-programmes/Suffolk-Diversity-Profile.pdf. Accessed 8 Jan 2019.

28. Office for National Statistics. **2011 rural/urban classification**. 2011. https://

For peer review only

For peer review only - http://bmjopen.bmj.com/site/about/guidelines.xhtml

www.ons.gov.uk/methodology/geography/geographicalproducts/ruralurbanclassifications/2011ruralurbanclassification. Accessed 8 Jan 2019.

29. Craggs C, van Sluijs EM, Corder K, Panter JR, Jones AP, Griffin SJ: **Do children's individual correlates of physical activity differ by home setting?** *Health Place.* 2011, **17**(5):1105-1112.

30. NHS Norfolk. **Annual Report by the Director of Public Health**. 2009.

31. Brown HE, Schiff A, van Sluijs EM: **Engaging families in physical activity research: A family-based focus group study**. *BMC Public Health.* 2015, **15**:1178-1186.

32. Stokols D: **Translating social ecological theory into guidelines for community health promotion**. *Am J Health Promot.* 1996, **10**(4):282-298.

33. Christensen P: **The health-promoting family: A conceptual framework for future research**. *Soc Sci Med.* 2004, **59**(2):377-387.

34. Deci EL, Ryan RM: **Intrinsic motivation and self-determination in human behavior**. New York: Springer Science & Business Media; 1985.

35. Morgan PJ, Young MD, Barnes AT, Eather N, Pollock ER, Lubans DR: **Engaging fathers to increase physical activity in girls: The "Dads And Daughters Exercising and Empowered"(DADEE) randomized controlled trial**. *Ann Behavl Med.* 2018, **53**:39-52.

36. Evenson KR, Catellier DJ, Gill K, Ondrak KS, McMurray RG: **Calibration of two objective measures of physical activity for children**. *J Sport Sci.* 2008, **26**(14):1557-1565.

37. Crouter SE, Horton M, Bassett Jr. DR: **Validity of ActiGraph Child-Specific Equations during Various Physical Activities**. *Med Sci Sport Exerc.* 2013, **45**(7):1403-1409.

38. Trost SG, Loprinzi PD, Moore R, Pfeiffer KA: **Comparison of accelerometer cut points for predicting activity intensity in youth**. *Med Sci Sport Exerc.* 2011, **43**(7):1360-1368.

39. Troiano RP, Berrigan D, Dodd KW, Mâsse LC, Tilert T, McDowell M: **Physical activity in the United States measured by accelerometer**. *Med Sci Sport Exerc.* 2008, **40**(1):181-188.

40. Collings PJ, Wijndaele K, Corder K, Westgate K, Ridgway CL, Dunn V, Goodyer I, Ekelund U, Brage S: **Levels and patterns of objectively-measured physical activity volume and**

For peer review only

For peer review only - http://bmjopen.bmj.com/site/about/guidelines.xhtml

intensity distribution in UK adolescents: The ROOTS study. *Int J Behav Nutr Phy.* 2014, **11**:23.

41. Pan H, Cole TJ: **LMSgrowth program version 2.77**. London, England: Child Growth Foundation; 2012.

42. Wareham NJ, Jakes RW, Rennie KL, Mitchell J, Hennings S, Day NE: **Validity and repeatability of the EPIC-Norfolk physical activity questionnaire**. *Int J Epidemiol.* 2002, **31**(1):168-174.

43. Corder K, van Sluijs EM, Wright A, Whincup P, Wareham NJ, Ekelund U: **Is it possible to assess free-living physical activity and energy expenditure in young people by self-report?** *Am J Clin Nutr.* 2009, **89**(3):862-870.

44. Lampard AM, Nishi A, Baskin ML, Carson TL, Davison KK: **The Activity Support Scale for Multiple Groups (ACTS-MG): Child-reported physical activity parenting in African American and non-Hispanic White families**. *Behav Med.* 2016, **42**(2):112-119.

45. van Sluijs EM, Skidmore PM, Mwanza K, Jones AP, Callaghan AM, Ekelund U, Harrison F, Harvey I, Panter J, Wareham NJ: **Physical activity and dietary behaviour in a population-based sample of British 10-year old children: The SPEEDY study (Sport, Physical activity and Eating behaviour: environmental Determinants in Young people)**. *BMC Public Health.* 2008, **8**(1):388.

46. The EuroQol Group: **EuroQol - a new facility for the measurement of health-related quality of life**. *Health Policy.* 1990, **16**(3):199-208.

47. Brooks R, The EuroQol Group: **EuroQol: The current state of play**. *Health Policy.* 1996, **37**(1):53-72.

48. Stevens KJ: **Working with children to develop dimensions for a preference-based, generic, pediatric, health-related quality-of-life measure**. *Qual Health Res.* 2010, **20**(3):340-351.

49. Stevens KJ: **Assessing the performance of a new generic measure of health-related quality of life for children and refining it for use in health state valuation**. *Appl Health Econ Health Pol.* 2011, **9**(3):157-169.

For peer review only

50. Corder K, van Sluijs EM, McMinn AM, Ekelund U, Cassidy A, Griffin SJ: **Perception versus reality: Awareness of physical activity levels of British children**. *Am J Prev Med.* 2010, **38**(1):1-8.

51. Godino JG, Watkinson C, Corder K, Sutton S, Griffin SJ, Van Sluijs EM: **Awareness of physical activity in healthy middle-aged adults: a cross-sectional study of associations with sociodemographic, biological, behavioural, and psychological factors**. *BMC Public Health* 2014, **14**:421.

52. D'Haese S, Gheysen F, De Bourdeaudhuij I, Deforche B, Van Dyck D, Cardon G: **The moderating effect of psychosocial factors in the relation between neighborhood walkability and children's physical activity**. *Int J Behav Nutr Phy.* 2016, **13**(1):128.

53. Motl RW, Dishman RK, Trost SG, Saunders RP, Dowda M, Felton G, Ward DS, Pate RR: **Factorial validity and invariance of questionnaires measuring social-cognitive determinants of physical activity among adolescent girls**. *Prev Med.* 2000, **31**(5):584-594.

54. Markland D, Tobin V: **A modification to the behavioural regulation in exercise questionnaire to include an assessment of amotivation**. *J Sport Exerc Psychol.* 2004, **26**(2):191-196.

55. Sebire SJ, Jago R, Fox KR, Edwards MJ, Thompson JL: **Testing a self-determination theory model of children's physical activity motivation: A cross-sectional study**. *Int J Behav Nutr Phy.* 2013, **10**:111.

56. Grotevant HD, Cooper CR: **Patterns of interaction in family relationships and the development of identity exploration in adolescence**. *Child Dev.* 1985, **56**(2):415-428.

57. Bengston PL, Grotevant HD: **The Individuality and Connectedness Q-sort: A measure for assessing individuality and connectedness in dyadic relationships**. *Pers Relationship.* 1999, **6**(2):213-225.

58. Beech BM, Klesges RC, Kumanyika SK, Murray DM, Klesges L, McClanahan B, Slawson D, Nunnally C, Rochon J, McLain-Allen B: **Child-and parent-targeted interventions: The Memphis GEMS pilot study**. *Ethnic Dis.* 2003, **13**(1; SUPP/1):S1-40.

For peer review only

For peer review only - http://bmjopen.bmj.com/site/about/guidelines.xhtml

59. Rhodes RE, Naylor P-J, McKay HA: **Pilot study of a family physical activity planning intervention among parents and their children**. *J Behav Med.* 2010, **33**(2):91-100.

60. Wilson EC: **A practical guide to value of information analysis**. *PharmacoEconomics* 2015, **33**(2):105-121.

61. Côté J, Salmela JH, Baria A, Russell S: **Organizing and interpreting unstructured qualitative data**. *Sport Psychol.* 1993, **7**:127-137.

62. National Institute for Health and Care Excellence: **Promoting physical activity for children and young people: Evidence update.** 2015. https://www.nice.org.uk/guidance/ph17/evidence/ph17-promoting-physical-activity-forchildren-and-young-people-evidence-update2. ccessed 8 Jan 2019.

For peer review only

For peer review only - http://bmjopen.bmj.com/site/about/guidelines.xhtml

Table 1.

Summary of FRESH Pilot Trial Intervention Components[1].

Intervention Components	Dose	Description	Behaviour Change Techniques	Targeted SDT constructs	Hypothesised mediators
1. 'Family time'	Minimum 1x/week, 10-20 mins	'Family time' is expected to provide a weekly (at minimum) opportunity for index children[2] and family members to review, revise, and update their family action planners. Family action planners prompt families to plan PA, monitor weekly steps, discuss any potential upcoming PA barriers and strategies to overcome them. Index children will be allocated as their family's 'team captain' leading in challenge selection and uploading steps on the FRESH website.	Goal-setting<br><br>Self-monitoring<br><br>Positive feedback on progress<br><br>Social support<br><br>Praise<br><br>Positive reinforcement	Perceived competence<br><br>Perceived relatedness<br><br>Perceived autonomy	Social support<br><br>Family social norms for PA<br><br>PA awareness<br><br>Basic needs satisfaction<br><br>PA motivation
2. FRESH website	Minimum 1x/week, 5-20 mins	The FRESH website will provide a place for families to self-monitor their step counts and set goals by selecting challenges of varying difficulty. Specifically, the website allows families to 'walk' around the world by choosing one of three target cities to 'walk to' weekly. The challenges are framed as an easy, moderate, or hard challenge, which represents a 0%, 5%, or 10% increase, respectively, relative to the average steps they take in preceding weeks. Once adults and children accumulate an average of 10,000 and 12,000 steps/day, the step challenge increases will be reduced to 0%, 2.5%, and 5%, respectively. Families will also have access to:<br>• a general resources area with suggestions for activities that families could do together.<br>• a map for a visual representation showing the locations families have travelled to.<br>• a step calculator that will convert activities not captured by	Goal-setting<br><br>Self-monitoring<br><br>Positive feedback on progress<br><br>Rewards	Perceived competence<br><br>Perceived relatedness<br><br>Perceived autonomy	Social support<br><br>Family social norms for PA<br><br>PA awareness<br><br>Basic needs satisfaction<br><br>PA motivation

For peer review only - http://bmjopen.bmj.com/site/about/guidelines.xhtml

		pedometers to steps (e.g., swimming).<br>• their families' **step history** by challenge.<br>• a **leader board**. Families will opt in to being included in a leader board that is updated weekly, where families get points for: selecting a challenge, uploading steps, completing a challenge, accumulating a family average of 10,000 steps/day over the week, increasing steps from the previous week, and going on streaks (e.g., 5 completed challenges in a row).<br>Families with an ongoing challenge will receive **email reminders** to log steps three days and one day before an impending challenge ends.			
3. Pedometry	Throughout intervention (8 weeks)	All family members will receive pedometers to enable self-monitoring and provide immediate feedback. To allow families to view their progress towards their proximal and distal step goals, they will be encouraged to log their steps onto the FRESH website and/or onto the family action planners.	Self-monitoring<br><br>Immediate feedback	Perceived competence<br><br>Perceived autonomy	Social support<br><br>Family social norms for PA<br><br>PA awareness<br><br>Basic needs satisfaction<br><br>PA motivation
4. Competence reinforcement/ rewards	~1x/week (8 weeks)	After completing a challenge or if the challenge week ended, to praise effort (i.e., competence reinforcement), children received **personalised supportive letters in the mail** and messages on the FRESH website.<br>They also received small online and **tangible rewards**.<br>• Online rewards will include virtual passport stamps (i.e., virtual rewards) and access to reinforcement materials (i.e., interactive information about the cities they have walked past during their challenge).<br>• **Tangible rewards** will be in the form of collectable FRESH cards, which display the city names corresponding with the families' challenge. The cards will also enable family to play a card game version of 'rock, paper, scissors'.<br>Children will receive 2-4 passport stamps/cards for completed challenges (i.e., as difficulty increased, more stamps were awarded) and 1 passport stamp/card for an incomplete challenge.	Feedback on progress<br><br>Rewards	Perceived competence	Basic needs satisfaction<br><br>PA awareness

For peer review only

Note. [1]This table has been adapted from our feasibility study [21]; bolded text indicates new elements added for the FRESH pilot trial (i.e., post-feasibility study). [2]The index child refers to the child aged 7-11 years in the family. **Abbreviations.** PA, physical activity; SDT, Self-Determination Theory.

For peer review only

Table 2.

Order of Measures and Estimated Duration of Data Collection[1,2].

Measure	Duration
1. Anthropometric measures (height, weight, waist circumference)	5 mins/person
2. Questionnaire	20 mins/family
3. Blood pressure[3]	10 mins/person
4. Step Test (aerobic fitness)	Prep: 5 mins/person<br>Test: 10 mins/family
5. Accelerometer and GPS explanation	5 mins/family
6. Fictional Family Holiday (family functioning)	10 mins/family
**Total duration of baseline measurement session (includes consent/assent process)**	**120 mins**
**Total duration of subsequent measurement sessions**	**105 mins**

Note. [1]This table has been adapted from our feasibility study [21]. [2]Estimate based on a four-person household, total time increases by ~30 minutes per additional family member. [3]Duration is halved when calculating total duration because multiple monitors will be used to enable two family members to be measured simultaneously.

For peer review only

For peer review only - http://bmjopen.bmj.com/site/about/guidelines.xhtml

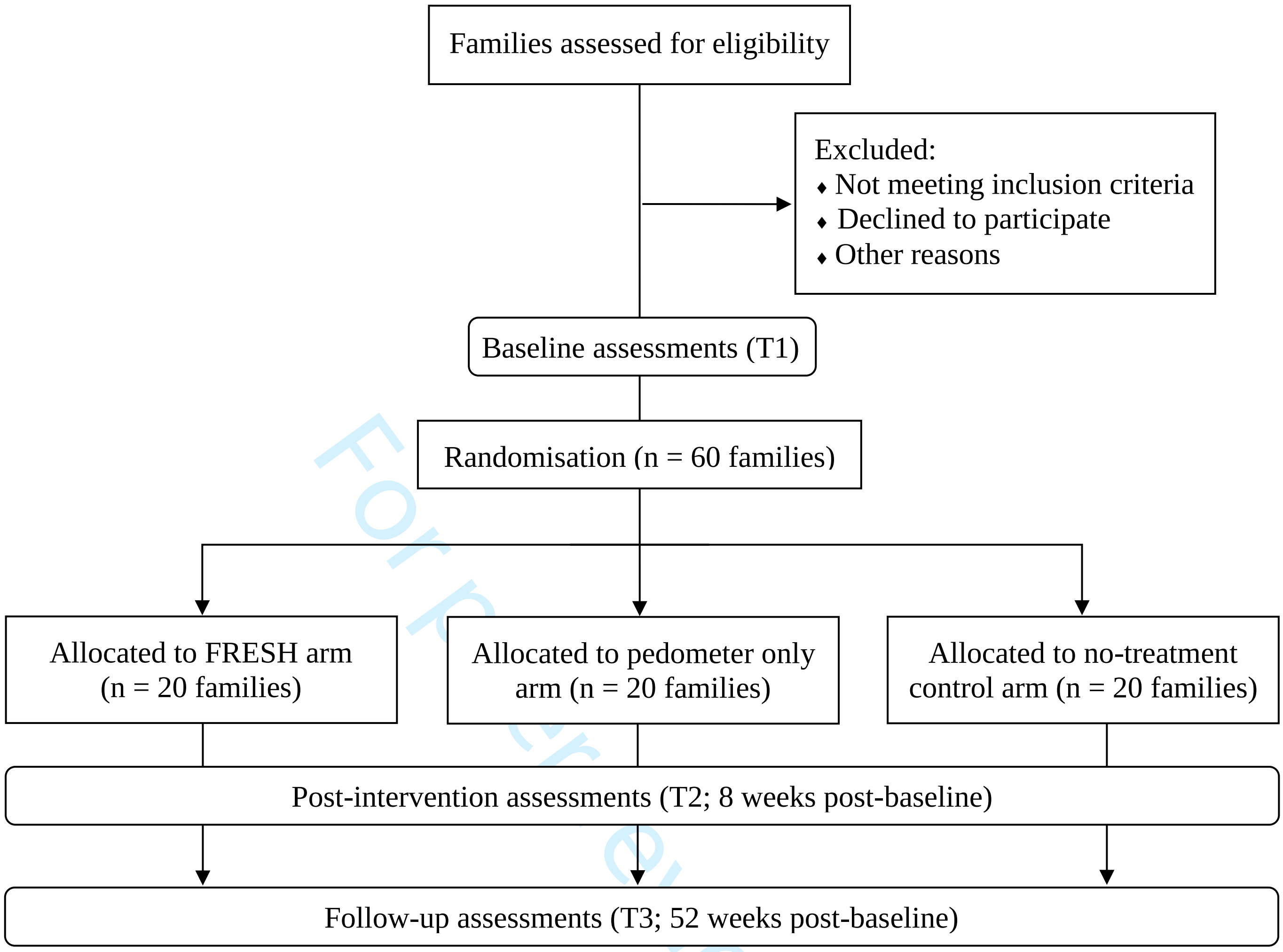

For peer review only

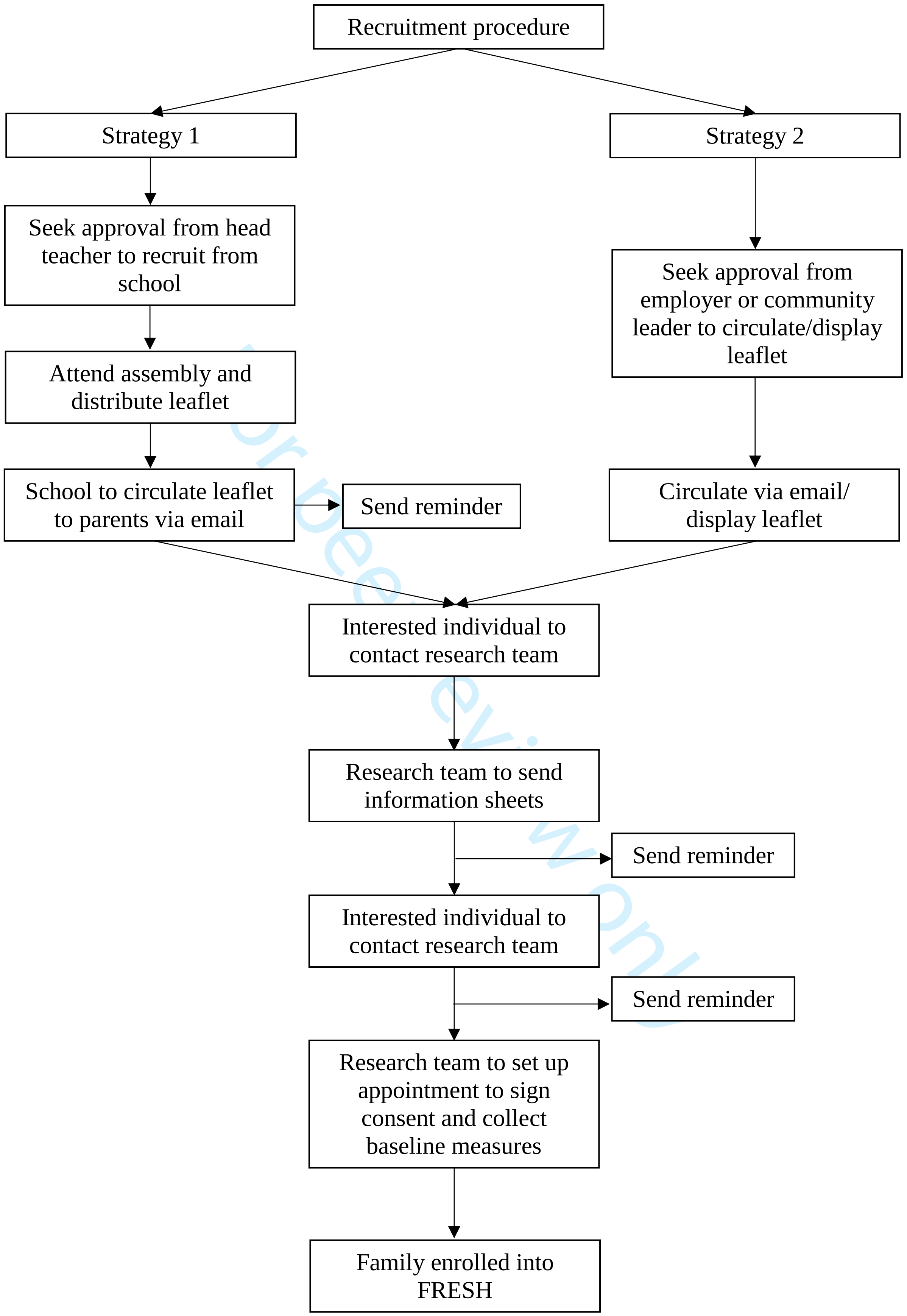
Recruitment procedure
Strategy 1
Strategy 2
Seek approval from head teacher to recruit from school
Seek approval from employer or community leader to circulate/display leaflet
Attend assembly and distribute leaflet
School to circulate leaflet to parents via email
Send reminder
Circulate via email/ display leaflet
Interested individual to contact research team
Research team to send information sheets
Send reminder
Interested individual to contact research team
Send reminder
Research team to set up appointment to sign consent and collect baseline measures
Family enrolled into FRESH

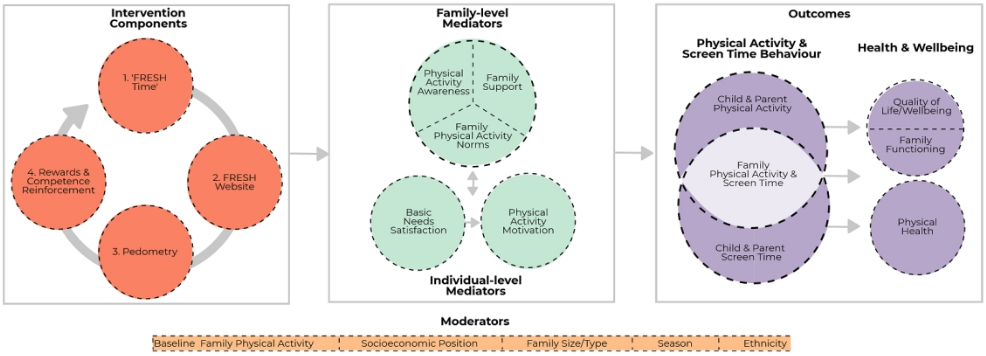

Figure 3. FRESH theoretical model.

SPIRIT

STANDARD PROTOCOL ITEMS: RECOMMENDATIONS FOR INTERVENTIONAL TRIALS

SPIRIT 2013 Checklist: Recommended items to address in a clinical trial protocol and related documents*

Section/item	Item No	Description	Reported on page No
**Administrative information**			
Title	1	Descriptive title identifying the study design, population, interventions, and, if applicable, trial acronym	1
Trial registration	2a	Trial identifier and registry name. If not yet registered, name of intended registry	2,14
	2b	All items from the World Health Organization Trial Registration Data Set	N/A
Protocol version	3	Date and version identifier	N/A
Funding	4	Sources and types of financial, material, and other support	15
Roles and responsibilities	5a	Names, affiliations, and roles of protocol contributors	1
	5b	Name and contact information for the trial sponsor	15
	5c	Role of study sponsor and funders, if any, in study design; collection, management, analysis, and interpretation of data; writing of the report; and the decision to submit the report for publication, including whether they will have ultimate authority over any of these activities	15
	5d	Composition, roles, and responsibilities of the coordinating centre, steering committee, endpoint adjudication committee, data management team, and other individuals or groups overseeing the trial, if applicable (see Item 21a for data monitoring committee)	N/A
**Introduction**			
Background and rationale	6a	Description of research question and justification for undertaking the trial, including summary of relevant studies (published and unpublished) examining benefits and harms for each intervention	4
	6b	Explanation for choice of comparators	N/A
Objectives	7	Specific objectives or hypotheses	4

For peer review only

Trial design	8	Description of trial design including type of trial (eg, parallel group, crossover, factorial, single group), allocation ratio, and framework (eg, superiority, equivalence, noninferiority, exploratory)	5
**Methods: Participants, interventions, and outcomes**			
Study setting	9	Description of study settings (eg, community clinic, academic hospital) and list of countries where data will be collected. Reference to where list of study sites can be obtained	5-6
Eligibility criteria	10	Inclusion and exclusion criteria for participants. If applicable, eligibility criteria for study centres and individuals who will perform the interventions (eg, surgeons, psychotherapists)	5
Interventions	11a	Interventions for each group with sufficient detail to allow replication, including how and when they will be administered	7-8, Table 1, Figure 3
	11b	Criteria for discontinuing or modifying allocated interventions for a given trial participant (eg, drug dose change in response to harms, participant request, or improving/worsening disease)	N/A
	11c	Strategies to improve adherence to intervention protocols, and any procedures for monitoring adherence (eg, drug tablet return, laboratory tests)	7-8, Table 1
	11d	Relevant concomitant care and interventions that are permitted or prohibited during the trial	
Outcomes	12	Primary, secondary, and other outcomes, including the specific measurement variable (eg, systolic blood pressure), analysis metric (eg, change from baseline, final value, time to event), method of aggregation (eg, median, proportion), and time point for each outcome. Explanation of the clinical relevance of chosen efficacy and harm outcomes is strongly recommended	9-11, Table 2
Participant timeline	13	Time schedule of enrolment, interventions (including any run-ins and washouts), assessments, and visits for participants. A schematic diagram is highly recommended (see Figure)	Figure 1
Sample size	14	Estimated number of participants needed to achieve study objectives and how it was determined, including clinical and statistical assumptions supporting any sample size calculations	12
Recruitment	15	Strategies for achieving adequate participant enrolment to reach target sample size	6-7, Figure 2
**Methods: Assignment of interventions (for controlled trials)**			
Allocation:			

For peer review only - http://bmjopen.bmj.com/site/about/guidelines.xhtml

Sequence generation	16a	Method of generating the allocation sequence (eg, computer-generated random numbers), and list of any factors for stratification. To reduce predictability of a random sequence, details of any planned restriction (eg, blocking) should be provided in a separate document that is unavailable to those who enrol participants or assign interventions	7
Allocation concealment mechanism	16b	Mechanism of implementing the allocation sequence (eg, central telephone; sequentially numbered, opaque, sealed envelopes), describing any steps to conceal the sequence until interventions are assigned	7
Implementation	16c	Who will generate the allocation sequence, who will enrol participants, and who will assign participants to interventions	7
Blinding (masking)	17a	Who will be blinded after assignment to interventions (eg, trial participants, care providers, outcome assessors, data analysts), and how	12
	17b	If blinded, circumstances under which unblinding is permissible, and procedure for revealing a participant's allocated intervention during the trial	12
**Methods: Data collection, management, and analysis**			
Data collection methods	18a	Plans for assessment and collection of outcome, baseline, and other trial data, including any related processes to promote data quality (eg, duplicate measurements, training of assessors) and a description of study instruments (eg, questionnaires, laboratory tests) along with their reliability and validity, if known. Reference to where data collection forms can be found, if not in the protocol	9-11, Table 2
	18b	Plans to promote participant retention and complete follow-up, including list of any outcome data to be collected for participants who discontinue or deviate from intervention protocols	7
Data management	19	Plans for data entry, coding, security, and storage, including any related processes to promote data quality (eg, double data entry; range checks for data values). Reference to where details of data management procedures can be found, if not in the protocol	N/A
Statistical methods	20a	Statistical methods for analysing primary and secondary outcomes. Reference to where other details of the statistical analysis plan can be found, if not in the protocol	12-13
	20b	Methods for any additional analyses (eg, subgroup and adjusted analyses)	N/A
	20c	Definition of analysis population relating to protocol non-adherence (eg, as randomised analysis), and any statistical methods to handle missing data (eg, multiple imputation)	N/A

For peer review only - http://bmjopen.bmj.com/site/about/guidelines.xhtml

**Methods: Monitoring**			
Data monitoring	21a	Composition of data monitoring committee (DMC); summary of its role and reporting structure; statement of whether it is independent from the sponsor and competing interests; and reference to where further details about its charter can be found, if not in the protocol. Alternatively, an explanation of why a DMC is not needed	N/A
	21b	Description of any interim analyses and stopping guidelines, including who will have access to these interim results and make the final decision to terminate the trial	N/A
Harms	22	Plans for collecting, assessing, reporting, and managing solicited and spontaneously reported adverse events and other unintended effects of trial interventions or trial conduct	N/A
Auditing	23	Frequency and procedures for auditing trial conduct, if any, and whether the process will be independent from investigators and the sponsor	N/A
**Ethics and dissemination**			
Research ethics approval	24	Plans for seeking research ethics committee/institutional review board (REC/IRB) approval	2, 14
Protocol amendments	25	Plans for communicating important protocol modifications (eg, changes to eligibility criteria, outcomes, analyses) to relevant parties (eg, investigators, REC/IRBs, trial participants, trial registries, journals, regulators)	N/A
Consent or assent	26a	Who will obtain informed consent or assent from potential trial participants or authorised surrogates, and how (see Item 32)	5, 7, 14
	26b	Additional consent provisions for collection and use of participant data and biological specimens in ancillary studies, if applicable	
Confidentiality	27	How personal information about potential and enrolled participants will be collected, shared, and maintained in order to protect confidentiality before, during, and after the trial	14
Declaration of interests	28	Financial and other competing interests for principal investigators for the overall trial and each study site	15
Access to data	29	Statement of who will have access to the final trial dataset, and disclosure of contractual agreements that limit such access for investigators	N/A
Ancillary and post-trial care	30	Provisions, if any, for ancillary and post-trial care, and for compensation to those who suffer harm from trial participation	N/A

For peer review only - http://bmjopen.bmj.com/site/about/guidelines.xhtml

Dissemination policy	31a	Plans for investigators and sponsor to communicate trial results to participants, healthcare professionals, the public, and other relevant groups (eg, via publication, reporting in results databases, or other data sharing arrangements), including any publication restrictions	14
	31b	Authorship eligibility guidelines and any intended use of professional writers	N/A
	31c	Plans, if any, for granting public access to the full protocol, participant-level dataset, and statistical code	N/A
**Appendices**			
Informed consent materials	32	Model consent form and other related documentation given to participants and authorised surrogates	N/A
Biological specimens	33	Plans for collection, laboratory evaluation, and storage of biological specimens for genetic or molecular analysis in the current trial and for future use in ancillary studies, if applicable	N/A

*It is strongly recommended that this checklist be read in conjunction with the SPIRIT 2013 Explanation & Elaboration for important clarification on the items. Amendments to the protocol should be tracked and dated. The SPIRIT checklist is copyrighted by the SPIRIT Group under the Creative Commons "Attribution-NonCommercial-NoDerivs 3.0 Unported" license.

For peer review only

BMJ Open

A whole family-based physical activity promotion intervention: The Families Reporting Every Step to Health (FRESH) pilot randomised controlled trial protocol

Journal:	*BMJ Open*
Manuscript ID	bmjopen-2019-030902.R1
Article Type:	Protocol
Date Submitted by the Author:	02-Jul-2019
Complete List of Authors:	Guagliano, Justin; University of Cambridge, MRC Epidemiology Unit and Centre for Diet and Activity Research<br>Brown, Helen; University of Cambridge, MRC Epidemiology Unit and Centre for Diet and Activity Research<br>Coombes, Emma; University of East Anglia, Norwich Medical School and UKCRC Centre for Diet and Activity Research<br>Haines, Elizabeth; University of Cambridge, MRC Epidemiology Unit and Centre for Diet and Activity Research<br>Hughes, Claire ; University of Cambridge, Centre for Family Research<br>Jones, Andrew; University of East Anglia, Norwich Medical School and UKCRC Centre for Diet and Activity Research<br>Morton, Katie; University of Cambridge, MRC Epidemiology Unit and UKCRC Centre for Diet and Activity Research<br>van Sluijs, Esther; University of Cambridge, MRC Epidemiology Unit and UKCRC Centre for Diet and Activity Research
**Primary Subject Heading**:	Public health
Secondary Subject Heading:	Public health
Keywords:	co-participation, co-physical activity, youth, parents

SCHOLARONE™ Manuscripts

A whole family-based physical activity promotion intervention: The Families Reporting Every Step to Health (FRESH) pilot randomised controlled trial protocol

Justin M. Guagliano[1§], Helen Elizabeth Brown[1], Emma Coombes[2], Elizabeth S. Haines[1], Claire Hughes[3], Andrew P. Jones[2], Katie L. Morton[1], & Esther M. F. van Sluijs[1]

[1]MRC Epidemiology Unit and UKCRC Centre for Diet and Activity Research, University of Cambridge, Cambridge, UK

[2]Norwich Medical School and UKCRC Centre for Diet and Activity Research, University of East Anglia, Norwich, UK

[3]Centre for Family Research, University of Cambridge, Cambridge, UK

[§]Corresponding author

Author email addresses:

JMG: Justin.Guagliano@mrc-epid.cam.ac.uk

HEB: Helen.Brown@bi.team

EC: Emma.Coombes@uea.ac.uk

ESH: Beth.Haines@mrc-epid.cam.ac.uk

CH: ch288@cam.ac.uk

APJ: A.P.Jones@uea.ac.uk

KLM: Katie.Morton@innoviatech.com

EvS: Esther.vanSluijs@mrc-epid.cam.ac.uk

For peer review only

For peer review only - http://bmjopen.bmj.com/site/about/guidelines.xhtml

Abstract

Introduction: Family-based physical activity (PA) interventions present a promising avenue to promote children's activity, however, high-quality experimental research is lacking. This paper describes the protocol for the FRESH (Families Reporting Every Step to Health) pilot trial, a child-led family-based PA intervention delivered online.

Methods and analysis: FRESH is a three-armed, parallel-group, randomised controlled pilot trial using a 1:1:1 allocation ratio with follow-up assessments at 8- and 52-weeks post-baseline. Families will be eligible if a minimum of one child in school Years 3-6 (aged 7-11 years) and at least one adult responsible for that child are willing to participate. Family members can take part in the intervention irrespective of their participation in the accompanying evaluation and vice versa.

Following baseline assessment, families will be randomly allocated to one of three arms: (1) FRESH, (2) pedometer-only, or (3) no-intervention control. All family members in the pedometer-only and FRESH arms receive pedometers and generic PA promotion information. FRESH families additionally receive access to the intervention website; allowing participants to select step challenges to 'travel' to target cities around the world, log steps, and track progress as they virtually globetrot. Control families will receive no treatment. All family members will be eligible to participate in the evaluation with two follow-ups (8 and 52 weeks). Physical (e.g., fitness, blood pressure), psychosocial (e.g., social support), and behavioural (e.g., objectively-measured family PA) measures will be collected each time point. At 8-week follow-up, a mixed-methods process evaluation will be conducted (questionnaires and family focus groups) assessing acceptability of the intervention and evaluation. FRESH families' website engagement will also be explored.

Ethics and dissemination: This study received ethical approval from the Ethics Committee for the School of the Humanities and Social Sciences at the University of Cambridge. Findings will be disseminated via peer-reviewed publications, conferences, and to participating families.

Trial registration number: This study was prospectively registered (ISRCTN12789422). Registered 16 March 2016. http://www.isrctn.com/ISRCTN12789422.

Keywords: youth, parent, mothers, fathers, mums, dads, co-participation, co-physical activity

For peer review only

For peer review only - http://bmjopen.bmj.com/site/about/guidelines.xhtml

Article Summary

Strengths and limitations of this study

- This pilot trial is among the first physical activity interventions that specifically targets and evaluates whole family engagement.
- Novel methodological work will be completed to compute family co-participation in physical activity using objective measures.
- The use of mixed-methods will provide unique insight and context for our quantitative findings.
- A long-term follow up (52 weeks post-baseline) will enable assessment of the potential for long-term effects and an accurate assessment of long-term participant retention, which is notoriously challenging in child-based research.
- However, this pilot trial will not allow us to draw conclusions of effectiveness.

For peer review only

For peer review only - http://bmjopen.bmj.com/site/about/guidelines.xhtml

Introduction

Although the benefits of regular physical activity in children are well-established [1, 2], roughly only half of UK children meet the recommended 60 minutes of daily moderate-to-vigorous intensity physical activity (MVPA) [3]. Moreover, observational data reveal that children are less active after school and at weekends compared to during school time, that children in rural areas are less active than their urban counterparts, and that MVPA declines steeply as children enter adolescence, particularly at weekends [4-7]. Intervening prior to adolescence may be important to help young people reach or maintain adequate physical activity levels.

Targeting families may therefore be one avenue to increase physical activity among children [8, 9]. In particular, parents can influence their children's health behaviours through a variety of mechanisms, including their general parenting style, parenting practices (e.g., rule setting, behavioural consequences, establishing behavioural expectations), and their control over the home environment [10, 11]. Parents can also act as gatekeepers to activity [12] and can play an important role in increasing their child's physical activity through role-modelling or parental support [13]. However, little physical activity promotion research has been conducted in the family compared to the school setting [14, 15]. Previous research suggests that: (1) involving family members is critical for sustained behaviour change [13, 16, 17]; and (2) home-based physical activity interventions that include the family are potentially more effective than those requiring the family to travel to community or other intervention locations [18, 19]. Many studies, however, only focus on promoting child physical activity instead of considering the family as a unit that may work together to change behaviour [20].

Our recent feasibility study [21] was among the first physical activity interventions to specifically target whole family engagement. The findings showed that it was feasible and acceptable to deliver and evaluate a family-targeted physical activity promotion intervention with high acceptability from participating families. Building upon this work, this paper describes the protocol for the Families Reporting Every Step to Health (FRESH) pilot trial. The aims of this pilot study are to: assess the feasibility and acceptability of the revised recruitment strategy, intervention, and outcome evaluation (after feasibility testing [21]); study long-term retention; and explore further intervention optimisation and preliminary effectiveness.

For peer review only - http://bmjopen.bmj.com/site/about/guidelines.xhtml

For peer review only

Methods and Analysis

The Standard Protocol Items: Recommendations for Interventional Trials [22] is used to guide the reporting of this study. We also use the Template for Intervention Description and Replication [23] to guide our description of the intervention.

Trial design

FRESH is a three-armed, parallel-group, randomised controlled pilot trial using a 1:1:1 allocation ratio with follow-up assessments at 8 and 52 weeks post-baseline (see Figure 1). Following baseline assessment, families will be randomly allocated to one of three arms: (1) FRESH arm, (2) pedometer-only arm, or (3) no-intervention control arm. All family members in the pedometer-only and FRESH arms receive pedometers and generic family physical activity promotion information (described later). FRESH families additionally receive access to the intervention website (described later). Control families will be asked to carry on as normal.

Participants

Families are deemed eligible to participate in this study if there is consent from at least one child in school Years 3-6 (aged 7-11 years, hereafter referred to as the index child) and at least one adult responsible for the index child. The adult must live with the index child in the main household (i.e., the index child's primary residence as indicated by the parent). There are no restrictions placed on family type (e.g., single parent, shared parenting, inclusion of extended family living in the main household) and no maximum number of participants per family. All participants need to be able to perform light-intensity physical activity (e.g., walking), have access to the Internet, and understand the English language sufficiently well to provide informed consent. Family members are able to take part in the intervention irrespective of their participation in the accompanying evaluation and vice versa. Exclusion criteria related to participation in specific assessments are outlined below.

Setting

For peer review only - http://bmjopen.bmj.com/site/about/guidelines.xhtml

Families will be recruited from rural Norfolk and Suffolk, counties in East Anglia, United Kingdom. Norfolk is 2,074 square miles in size and had a total estimated population of 898,400 in 2017 (most recent estimate) [24] and Suffolk is 1,466 square miles and had a total estimated population of 756,978 in 2017 (most recent estimate) [25]. According to the Norfolk and Suffolk County Councils [26, 27], approximately 53% and 42% of the Norfolk and Suffolk populations respectively are classified as living in a rural area. Based on the Office for National Statistics [28] classification, 'rural' will be defined as having a postcode falling in a small town, village, hamlet, or dispersed settlement. In both counties, existing inequalities have been identified, including physical activity, obesity and other indicators of child ill health, school readiness, and attainment [6, 29, 30].

Recruitment

The recruitment of families is known to be particularly challenging [11, 13] and we have described specific recruitment challenges we have encountered previously [21]. To overcome these challenges, we will use a multi-faceted recruitment strategy, including targeting adults (parents) directly, and messaging that focuses on the wider benefits of research participation (e.g., spending more time together as a family) as opposed to solely focusing on increasing physical activity or obesity prevention [31]. Recruitment will be undertaken over an estimated 3 months (with a recruitment rate of ~20 families/month), using two main strategies that target adults and children, as summarised in Figure 2. The first strategy involves recruitment in the school setting and the second in employer-based and community-based settings (e.g., Brownies/Cubs, community centres, GP surgeries). Alternative recruitment settings may also be explored (e.g., online/traditional media) if needed, and will follow the same procedure as the second strategy. For logistical purposes, we aim to find recruitment settings located roughly within an hour commute of Cambridge, United Kingdom.

Recruitment protocol. To recruit schools, employers, and community-based organisations, we will aim to first contact those in lead positions (e.g., head teachers, human resources, health and wellbeing leaders, heads of community-based organisations). An information pack detailing the purpose of the study and all procedures will be included in our correspondence with lead personnel. Also included in the information pack and recruitment material will be a link to a recruitment video,

For peer review only

which was developed with families following the suggestion of participants in the FRESH feasibility study [21]. For school-based recruitment, verbal or written approval will be sought to send home study leaflets with children, circulate our leaflet to parents online, and send an online reminder to parents approximately two weeks later. We will also seek permission to present to Key Stage 2 students (Years 3-6; 7-11 years) at a scheduled school assembly. Similarly, for employer- and community-based recruitment, we will seek approval to circulate our study information to employees or members either online or hardcopy. In all cases, interested parents are asked to express interest by contacting the study team via e-mail or a free-to-call telephone number, after which eligibility will be assessed and study information emailed. A baseline assessment appointment will be made with families still interested in participating. Written informed consent will be obtained for all participating adults and written parental consent and child assent for each participating child during this appointment, prior to baseline assessments.

Retention. To encourage retention, we will remain in regular contact with all participating families (through intervention website, newsletter/holiday cards), and offer measurement incentives and study feedback. Each individual participant will receive a £5 voucher upon return of the accelerometer and GPS monitors at each measurement time point. Retention will be monitored by study group and demographic characteristics to observe whether differences in retention occur.

Randomisation

Randomisation will occur after baseline assessments and the unit of randomisation will be at the family level (i.e., the index child and all participating family members). Families will be randomised in blocks of six by an independent statistician using a computer generated algorithm produced with Stata (version 14; StataCorp. TX: StataCorp LP) and stratified by county (i.e., Norfolk or Suffolk). The random allocation sequence will be implemented via a database created in-house on Microsoft Access by independent data management staff. A study coordinator will use the database after baseline to determine which study arm a family is allocated to. No one directly involved in the evaluation will have access to the allocation code or complete sequence.

For peer review only - http://bmjopen.bmj.com/site/about/guidelines.xhtml

Intervention protocol for FRESH arm families

The development, feasibility, acceptability, and refinements made to the intervention prior to the current pilot trial have been previously described [21]. In brief, the intervention the FRESH arm will receive is primarily a goal-setting and self-monitoring intervention, delivered online, aimed at increasing physical activity in whole families. The intervention uses concepts from the Socio-Ecological Model [32] and Family Systems Theory [33] and operationalises constructs from Self-Determination Theory [34] to inform behaviour change strategies. A detailed description of the FRESH intervention components, behaviour change techniques, targeted theoretical constructs, and hypothesised mediators are in Table 1. Additionally, the FRESH logic model can be found in Figure 3.

A week after baseline measures, each family allocated to the FRESH arm will have an hour-long kick-off meeting, scheduled in the family home (or an alternative location can be arranged) with a facilitator. The facilitator will introduce families to the intervention components, accompanying materials (e.g., family action planner), and distribute pedometers. Families will also receive their first of four pieces of generic walking information (described in a subsequent section). However, the main goals of this meeting are to familiarise families with the intervention website and their pedometers and to prompt weekly 'family time' meetings (described in Table 1) in which they complete their action planners and select a new challenge city to 'walk to' on the FRESH intervention website. All families will receive a follow-up phone call a week after their kick-off meeting to discuss any issues or ask any clarifying questions. Participant-initiated distant support will continue to be available, where participants can contact the research team with questions or to report issues (e.g., website bugs, pedometer issues).

Table 1 provides a detailed description of the FRESH intervention components. In each family, the index child (or children, if multiple) will be designated the role of 'team captain(s)', which involves taking the lead in selecting challenges and uploading steps online. Evidence suggests that children may act as change agents to elicit changes to the psychosocial environment [14]; therefore, promoting the index children to the role of family 'team captain(s)' may strengthen child buy-in, perceived autonomy, and improve intervention fidelity. All family members will be given pedometers,

For peer review only - http://bmjopen.bmj.com/site/about/guidelines.xhtml

wearing them for as long as possible daily, to capture their steps during challenges. Pedometers are simple to use, convenient, and are associated with effective interventions for increasing parent-child physical activity [e.g., 35]. After the challenge week is over, whether a family completes their challenge or not, they will receive personalised competence reinforcement messages praising their effort online and hardcopy letters (addressed and mailed to all participating children in the family). In addition, online and tangible rewards will be given to participating children after a challenge week. If a family does not complete a challenge, they will progress to a hidden city along their challenge route, as opposed to the city they chose, and will still receive a reinforcement message and reward, as described above. Families with an ongoing challenge will receive email reminders to log steps three days and one day before an impending challenge ends. After every challenge week, the above cycle will repeat starting with the next 'family time' meeting. Following assessments at 8 weeks post-baseline, families retain access to the website and their pedometers, and can continue using it for as long as they like. There will also be continued support in terms of website updates (e.g., leaderboard and parental resource updates) and participants will continue to receive competence reinforcement letters and rewards.

Protocol for pedometer-only and control families

Following the baseline assessment, families allocated to the pedometer-only arm will be mailed pedometers and generic family physical activity promotion information produced by Walk4Life, a sub-brand of Change4Life (www.nhs.uk/change4life). Information will continue to be emailed to families (pedometer-only and FRESH arm families) fortnightly on four occasions. The information will provide families with tips to get walking daily and games that can be played while walking. Control families will be asked to carry on as normal and will not receive access to the intervention website, pedometers, or any generic information.

Outcome evaluation measures

Table 2 outlines the measures taken, including assessment order and estimated duration. Data collection will be carried out by two trained research staff and will occur in participating families'

For peer review only - http://bmjopen.bmj.com/site/about/guidelines.xhtml

homes (or an alternative location by arrangement). Outcomes will be assessed at baseline (prior to randomisation; spring/summer 2018), 8 weeks post-baseline (summer/autumn 2018), and follow up (52 weeks post-bas`eline; spring/summer 2019) on all consenting family members (excluding children ≤ 2 years).

Primary outcome

Physical activity outcomes. To assess physical activity, participants will be asked to simultaneously wear an ActiGraph GT3X+ tri-axial accelerometer (ActiGraph LLC; Pensacola, Florida) and QStarz Travel Recorder BT1000X global positioning system (GPS) monitor (QStarz; Taipei, Taiwan). The accelerometer will be initialised to record data at a sampling rate of 50 Hz, while the GPS will be set to record a location every 10 seconds. The devices will be worn on each hip during waking hours for 7 consecutive days. A valid week will be defined as a minimum of 420 minutes/day from 3 days over the 7-day measurement period; however, this definition may be altered depending on participant compliance. Non-wear will be defined as ≥ 90 mins consecutive zeros using the vector magnitude. Raw accelerometer counts will be downloaded and integrated into 5-second epochs. Evenson et al. cut-points [36] have been recommended to estimate physical activity intensity in youth [37, 38] and Troiano et al. [39] cut-points for adults, these cut-points will be used in this study. In addition, family co-participation in physical activity will be measured by matching accelerometer and GPS data using Java. Novel methodological work will be completed to compute minutes of physical activity per day when family members are together at a given location from the matched data.

For peer review only

Secondary outcomes

Health outcomes. Aerobic fitness will be measured using an 8-minute submaximal step test (with 2-minute rest), which provides an individual calibration of heart rate to work rate (energy expenditure per unit time) to predict a fitness estimate of a participants' heart rate recovery index [40]. Children < 7 years will be excluded from the aerobic fitness test.

Height and weight will be measured once with a Leicester portable stadiometer and Seca 877 digital scale, respectively. Waist circumference will be measured twice, using a non-elastic tape measure. A third measure is taken if the first two measures differed by ≥ 3 cm. Body mass index will

For peer review only - http://bmjopen.bmj.com/site/about/guidelines.xhtml

be calculated, and converted into age- and sex-specific percentiles using standard growth charts for children [41].

Behavioural and psychosocial measures. Behavioural and psychosocial measures will be measured via questionnaires distributed to adults and children (those ≤ 4 years will not complete questionnaires). The questionnaires can be viewed in the Supplementary File. These will include: adult and child screen-time use [42-45]; quality of life [46-49]; family co-participation in physical activity [45]; physical activity awareness [50, 51]; family social norms for physical activity [52, 53]; family support [52]; children's and adult's motivation for physical activity [54, 55]; and children's perceived autonomy, competence, and relatedness [55].

Family functioning. The Fictional Family Holiday Paradigm will be used to assess family functioning via family relationships [56] and connectedness [57]. In this observational paradigm, each family is asked to spend 10 minutes planning and discussing a week-long holiday itinerary with unlimited budget. The video-recorded activity is then transcribed prior to coding family functioning, which is indexed through multiple conversational markers, including: 'power sharing' (i.e., taking turns); parental elicitation of all family members' viewpoints; expressions of individuality (e.g., suggestions for destinations/activities or disagreements) and connectedness (e.g., agreements, questions, or initiating compromise) [57]; as well as non-verbal markers, including expressions of amity (e.g., laughter, displays of affection) or hostility (e.g., sarcasm, anger).

Family out-of-pocket expenditure for physical activity. Physical activity related expenditure for each family member will be collected via a questionnaire that was developed and refined following our feasibility study [21]. The questionnaire comprises two questions about expenditure related to membership fees and subscriptions (e.g., for sports clubs, fitness centres) and sports equipment (e.g., sportswear, gadgets) and is completed by the same adult at each time point for their whole family.

Process evaluation

A mixed-methods process evaluation will be conducted after assessments 8-weeks post-baseline. Using opened-ended and 4-point Likert-scale questions (1 = strongly disagree, 4 = strongly agree),

For peer review only - http://bmjopen.bmj.com/site/about/guidelines.xhtml

adults will self-report their overall opinion of FRESH, the intervention components, measurements, and suggestions for improvement. Children will also self-report on the above topics, responding to dichotomous 'yes/no' questions. In addition, semi-structured focus groups will be conducted online on 10/20 FRESH families and 5/20 families from each of the other two arms (i.e., pedometer-only, control). Focus groups will focus on families' experience taking part in the trial, perceived acceptability of individual intervention components, intervention fidelity, challenges/barriers encountered, and suggested improvements, as appropriate based on study arm allocation. All focus groups will be transcribed verbatim. We will also explore FRESH families' engagement with the intervention website (e.g., page views, challenges accepted/completed) and aspects of the recruitment process (e.g., recruitment duration, resources used, comparisons of recruitment strategies).

Patient and public involvement

FRESH was developed with substantial input from children and families from the public, which has been described elsewhere [21]. Since the completion of the FRESH feasibility study, families from the public have been further involved with the optimisation of FRESH in a number of ways. As mentioned above, we sought the involvement of families from the public to develop a recruitment video; these families helped develop the script and acted in the video, which can be viewed here: www.youtube.com/watch?v=UxUHN1JsjUM. We also asked families to engage with the FRESH website and provide feedback to inform modifications that could be made. Lastly, we have included parents as members on our Study Steering Committee.

Blinding

Researchers conducting analyses will be blinded to family treatment allocation. Research assistants will not be explicitly told family treatment allocation. However, it will not be possible to conceal treatment allocation from participating families.

Sample size considerations

For peer review only - http://bmjopen.bmj.com/site/about/guidelines.xhtml

Since this is a pilot study, a sample size calculation will not be performed. We plan to recruit a sample of 60 families, with a sample size of ~180-240 participants based on ~3-4 members per family. Our estimated sample size is based on prior study experience [31] and sample sizes of previous pilot studies [58, 59].

Data analysis

Quantitative data. Statistical analyses of the primary and applicable secondary outcomes will be conducted using linear mixed models in Stata (version 14; StataCorp. TX: StataCorp LP). The models will be used to assess the impact of treatment (FRESH, pedometer-only, or control), time, (baseline, 8-weeks, and 52-weeks), and the group-by-time interaction. An estimate of effect and 95% confidence interval will be calculated. Descriptive statistics will also be calculated to describe data related to recruitment, retention, acceptability, family functioning, and website engagement.

Economic analyses. Resource use counts (e.g. time spent training families) will be converted to cost using unit costs from a common price year, and adjusted to the common price year using the consumer price index. Total cost per family will be the sum of intervention delivery and physical activity expenses in each arm. Incremental cost per family at each time point will be combined with change in MVPA to calculate a measure of cost-effectiveness. Analysis of uncertainty will include reporting 95% confidence intervals around increments and the cost-effectiveness acceptability curve, showing the probability of cost-effectiveness as a function of willingness to pay for an hour of MVPA (taking account of dominance and extended dominance as appropriate). The incremental cost per quality-adjusted life year (QALY) of the FRESH interventions over a 10 year horizon will also be calculated. The emphasis of these analyses will not be on the point estimate means, but on identifying the uncertainty in cost-effectiveness, informing a value of information analysis [60]. The value of information analysis will be conducted to predict the efficient sample size of the definitive FRESH trial as a function of *willingness to pay for an additional hour of MVPA*, and, using the modelled results, predict the efficient sample size as a function of *willingness to pay* for a QALY.

Qualitative data. A content analysis will be conducted using existing guidelines [61] to explore the feasibility and acceptability of the revised FRESH intervention, outcome evaluation, and

For peer review only - http://bmjopen.bmj.com/site/about/guidelines.xhtml

suggestions for further intervention optimisation via family focus groups. Specifically, the analysis will be conducted in two separate phases. During the data organisation phase, text from each transcript will be divided into segments (meaning units) to produce a set of concepts that reflected meaningful pieces of information [61]. Tags will then be assigned to each meaning unit. Tagging will be performed by one researcher, with a second double-tagging ~25% of transcripts. For the data interpretation phase, the inventory of tags from all transcripts will examined by two researchers, which will lead to the emergence of themes and sub-themes within each overarching category.

Discussion

The FRESH study will provide a response to calls for the need for innovative interventions targeting young people and families [62] and builds upon our previous work [21]. As far as we are aware, FRESH is among the first physical activity interventions that specifically targets and evaluates whole family engagement.

The overall aim of a future definitive trial will be to establish the long-term effectiveness and cost-effectiveness of the family-based FRESH intervention to promote MVPA in young people and their families. A decision to progress to a definitive trial will be made with an independent Study Steering Committee, using the following parameters and taking into account qualitative findings on the acceptability of trial procedures:

- Demonstrable feasibility of recruiting 20 families/month (accounting for increased staffing in a future definitive trial) and retaining 75% of index children at follow-up (52 weeks post-baseline);
- Good intervention adherence for families in the FRESH arm, defined as >75% of families uploading steps at least 6 times in the first 3 months of the study;
- Intervention optimisation is feasible (i.e., identified adaptations are practical, affordable, acceptable);
- Evidence to suggest an adequately powered trial would require a feasible number of participants;
- Discontinuation of trial arm based on evidence of harm or limited acceptability/feasibility;

For peer review only - http://bmjopen.bmj.com/site/about/guidelines.xhtml

- Positive expected net gain of sampling from definitive trial.

Ethics and dissemination

Written informed consent will be obtained for all participating adults and written parental consent and child assent for each participating child prior to collecting baseline assessments. Ethical approval has been obtained from the Ethics Committee for the School of the Humanities and Social Sciences at the University of Cambridge (ID number: 17/113) and this study has been prospectively registered (ISRCTN12789422).

For peer review only

For peer review only - http://bmjopen.bmj.com/site/about/guidelines.xhtml

List of abbreviations

FRESH, Families Reporting Every Step to Health; GPS, global positioning system; MVPA, moderate-to-vigorous physical activity.

Declarations

Authors' contributions. EvS (principal investigator), HEB, CH, APJ, and KLM secured funding for the research. All authors contributed to the study design. JMG drafted the manuscript. HEB, EC, ESH, CH, APJ, KLM, and EvS critically reviewed and revised the manuscript. All authors read and approved the final manuscript.

Funding. This work was supported by the National Institute for Health Research Public Health Research Programme (project number 15/01/19). Intervention costs for the current study were supported by Active Norfolk and Suffolk County Council. Funding was also received from the Medical Research Council (project number MC_UU_12015/7).

Competing interests. The authors declare that they have no competing interest. The views expressed are those of the author(s) and not necessarily those of the National Health Service, the National Institute for Health Research, or the Department of Health and Social Care.

Acknowledgements. This work was undertaken by the Centre for Diet and Activity Research (CEDAR), where funding from Cancer Research UK, the British Heart Foundation, the Economic and Social Research Council, the Medical Research Council, the National Institute for Health Research, and the Wellcome Trust, under the auspices of the UK Clinical Research Collaboration, is gratefully acknowledged [087636/Z/08/Z; ES/G007462/1; MR/K023187/1].

For peer review only - http://bmjopen.bmj.com/site/about/guidelines.xhtml

Figure legend.

Figure 1. Participant flow diagram.

Figure 2. Recruitment strategy.

Figure 3. FRESH theoretical model.

For peer review only

For peer review only - http://bmjopen.bmj.com/site/about/guidelines.xhtml

References

1. Janssen I, LeBlanc AG: **Systematic review of the health benefits of physical activity and fitness in school-aged children and youth**. *Int J Behav Nutr Phy.* 2010, **7**(40):1-16.

2. Poitras VJ, Gray CE, Borghese MM, Carson V, Chaput J-P, Janssen I, Katzmarzyk PT, Pate RR, Connor Gorber S, Kho ME: **Systematic review of the relationships between objectively measured physical activity and health indicators in school-aged children and youth**. *Appl Physiol Nutr Me.* 2016, **41**(6):S197-S239.

3. Griffiths LJ, Cortina-Borja M, Sera F, Pouliou T, Geraci M, Rich C, Cole TJ, Law C, Joshi H, Ness AR: **How active are our children? Findings from the Millennium Cohort Study**. *BMJ Open.* 2013, **3**(8):e002893.

4. Brooke HL, Atkin AJ, Corder K, Ekelund U, van Sluijs EM: **Changes in time-segment specific physical activity between ages 10 and 14 years: A longitudinal observational study**. *J Sci Med Sport.* 2016, **19**(1):29-34.

5. Corder K, Craggs C, Jones AP, Ekelund U, Griffin SJ, van Sluijs EM: **Predictors of change differ for moderate and vigorous intensity physical activity and for weekdays and weekends: A longitudinal analysis**. *Int J Behav Nutr Phy.* 2013, **10**:69.

6. Corder K, Sharp S, Atkin AJ, Griffin S, Jones A, Ekelund U, van Sluijs E: **Change in objectively measured physical activity during the transition to adolescence**. *Brit J Sport Med.* 2013, **49**(11):730-736.

7. Hallal PC, Andersen LB, Bull FC, Guthold R, Haskell W, Ekelund U: **Global physical activity levels: surveillance progress, pitfalls, and prospects**. *Lancet.* 2012, **380**(9838):247-257.

8. Kaushal N, Rhodes RE: **The home physical environment and its relationship with physical activity and sedentary behavior: A systematic review**. *Prev Med.* 2014, **67**:221-237.

9. Maitland C, Stratton G, Foster S, Braham R, Rosenberg M: **A place for play? The influence of the home physical environment on children's physical activity and sedentary behaviour**. *Int J Behav Nutr Phy.* 2013, **10**(1):99.

For peer review only

For peer review only - http://bmjopen.bmj.com/site/about/guidelines.xhtml

10. Ventura AK, Birch LL: **Does parenting affect children's eating and weight status?** *Int J Behav Nutr Phy.* 2008, **5**(15).

11. Morgan PJ, Jones RA, Collins CE, Hesketh KD, Young MD, Burrows TL, Magarey AM, Brown HL, Hinkley T, Perry RA: **Practicalities and research considerations for conducting childhood obesity prevention interventions with families**. *Children.* 2016, **3**(4):24-40.

12. Patrick H, Hennessy E, McSpadden K, Oh A: **Parenting styles and practices in children's obesogenic behaviors: scientific gaps and future research directions**. *Child Obes.* 2013, **9**(S1):S73-S86.

13. O'Connor TM, Jago R, Baranowski T: **Engaging parents to increase youth physical activity: A systematic review**. *Am J Prev Med.* 2009, **37**(2):141-149.

14. Brown HE, Atkin AJ, Panter J, Wong G, Chinapaw MJ, van Sluijs E: **Family-based interventions to increase physical activity in children: A systematic review, meta-analysis and realist synthesis**. *Obes Rev.* 2016, **17**(4):345-360.

15. Marsh S, Foley L, Wilks D, Maddison R: **Family-based interventions for reducing sedentary time in youth: A systematic review of randomized controlled trials**. *Obes Rev.* 2014, **15**(2):117-133.

16. van Sluijs EM, McMinn A: **Preventing obesity in primary schoolchildren**. *BMJ.* 2010, **340**:819.

17. Kipping RR, Howe LD, Jago R, Campbell R, Wells S, Chittleborough CR, Mytton J, Noble SM, Peters TJ, Lawlor DA: **Effect of intervention aimed at increasing physical activity, reducing sedentary behaviour, and increasing fruit and vegetable consumption in children: Active for Life Year 5 (AFLY5) school based cluster randomised controlled trial**. *BMJ.* 2014, **348**:348-361.

18. van Sluijs EM, Kriemler S, McMinn AM: **The effect of community and family interventions on young people's physical activity levels: A review of reviews and updated systematic review**. *Brit J Sport Med.* 2011, **45**(11):914-922.

For peer review only

For peer review only - http://bmjopen.bmj.com/site/about/guidelines.xhtml

19. Rhodes RE, Lim C: **Promoting parent and child physical activity together: elicitation of potential intervention targets and preferences**. *Health Educ Behav.* 2018, **45**(1):112-123.

20. Shonkoff JP, Fisher PA: **Rethinking evidence-based practice and two-generation programs to create the future of early childhood policy**. *Dev Psychopathol.* 2013, **25**(4 pt 2):1635-1653.

21. Guagliano JM, Brown HE, Coombes E, Hughes C, Jones AP, Morton KL, Wilson EC, van Sluijs EM: **The development and feasibility of a randomised family-based physical activity promotion intervention: the Families Reporting Every Step to Health (FRESH) study**. *Pilot and Feasibility Studies.* 2019, **5**:21.

22. Chan A-W, Tetzlaff JM, Altman DG, Laupacis A, Gøtzsche PC, Krleža-Jerić K, Hróbjartsson A, Mann H, Dickersin K, Berlin JA: **SPIRIT 2013 statement: Defining standard protocol items for clinical trials**. *Ann Intern Med* 2013, **158**(3):200-207.

23. Hoffmann TC, Glasziou PP, Boutron I, Milne R, Perera R, Moher D, Altman DG, Barbour V, Macdonald H, Johnston M: **Better reporting of interventions: template for intervention description and replication (TIDieR) checklist and guide**. *BMJ.* 2014, **348**:g1687.

24. Norfolk County Council. **Data and information about Norfolk's population.** 2015. http://www.norfolkinsight.org.uk. Accessed 8 Jan 2019.

25. Suffolk Observatory. **Population estimates.** 2018. https://www.suffolkobservatory.info/population/report/view/0c18eadb6fb544c1bee07bc7561 8ec49/E10000029/. Accessed 8 Jan 2019.

26. Norfolk County Council. **Norfolk Rural Development Strategy 2013-2020.** 2013. https://www.norfolk.gov.uk/-/media/norfolk/downloads/what-we-doand-how-we-work/policy-performance-and-partnerships/policies-andstrategies/business/norfolk-rural-development-strategy-2013-20.pdf. Accessed 8 Jan 2019.

27. Suffolk County Council. **Suffolk Demographic Profile**. 2015. https://www.suffolk.gov.uk/assets/council-and-democracy/our-aims-and-transformation-programmes/Suffolk-Diversity-Profile.pdf. Accessed 8 Jan 2019.

28. Office for National Statistics. **2011 rural/urban classification**. 2011. https://

For peer review only

For peer review only - http://bmjopen.bmj.com/site/about/guidelines.xhtml

www.ons.gov.uk/methodology/geography/geographicalproducts/ruralurbanclassifications/2011ruralurbanclassification. Accessed 8 Jan 2019.

29. Craggs C, van Sluijs EM, Corder K, Panter JR, Jones AP, Griffin SJ: **Do children's individual correlates of physical activity differ by home setting?** *Health Place.* 2011, **17**(5):1105-1112.

30. NHS Norfolk. **Annual Report by the Director of Public Health**. 2009.

31. Brown HE, Schiff A, van Sluijs EM: **Engaging families in physical activity research: A family-based focus group study**. *BMC Public Health.* 2015, **15**:1178-1186.

32. Stokols D: **Translating social ecological theory into guidelines for community health promotion**. *Am J Health Promot.* 1996, **10**(4):282-298.

33. Christensen P: **The health-promoting family: A conceptual framework for future research**. *Soc Sci Med.* 2004, **59**(2):377-387.

34. Deci EL, Ryan RM: **Intrinsic motivation and self-determination in human behavior**. New York: Springer Science & Business Media; 1985.

35. Morgan PJ, Young MD, Barnes AT, Eather N, Pollock ER, Lubans DR: **Engaging fathers to increase physical activity in girls: The "Dads And Daughters Exercising and Empowered"(DADEE) randomized controlled trial**. *Ann Behavl Med.* 2018, **53**:39-52.

36. Evenson KR, Catellier DJ, Gill K, Ondrak KS, McMurray RG: **Calibration of two objective measures of physical activity for children**. *J Sport Sci.* 2008, **26**(14):1557-1565.

37. Crouter SE, Horton M, Bassett Jr. DR: **Validity of ActiGraph Child-Specific Equations during Various Physical Activities**. *Med Sci Sport Exerc.* 2013, **45**(7):1403-1409.

38. Trost SG, Loprinzi PD, Moore R, Pfeiffer KA: **Comparison of accelerometer cut points for predicting activity intensity in youth**. *Med Sci Sport Exerc.* 2011, **43**(7):1360-1368.

39. Troiano RP, Berrigan D, Dodd KW, Mâsse LC, Tilert T, McDowell M: **Physical activity in the United States measured by accelerometer**. *Med Sci Sport Exerc.* 2008, **40**(1):181-188.

40. Collings PJ, Wijndaele K, Corder K, Westgate K, Ridgway CL, Dunn V, Goodyer I, Ekelund U, Brage S: **Levels and patterns of objectively-measured physical activity volume and**

For peer review only

For peer review only - http://bmjopen.bmj.com/site/about/guidelines.xhtml

intensity distribution in UK adolescents: The ROOTS study. *Int J Behav Nutr Phy.* 2014, **11**:23.

41. Pan H, Cole TJ: **LMSgrowth program version 2.77**. London, England: Child Growth Foundation; 2012.

42. Wareham NJ, Jakes RW, Rennie KL, Mitchell J, Hennings S, Day NE: **Validity and repeatability of the EPIC-Norfolk physical activity questionnaire**. *Int J Epidemiol.* 2002, **31**(1):168-174.

43. Corder K, van Sluijs EM, Wright A, Whincup P, Wareham NJ, Ekelund U: **Is it possible to assess free-living physical activity and energy expenditure in young people by self-report?** *Am J Clin Nutr.* 2009, **89**(3):862-870.

44. Lampard AM, Nishi A, Baskin ML, Carson TL, Davison KK: **The Activity Support Scale for Multiple Groups (ACTS-MG): Child-reported physical activity parenting in African American and non-Hispanic White families**. *Behav Med.* 2016, **42**(2):112-119.

45. van Sluijs EM, Skidmore PM, Mwanza K, Jones AP, Callaghan AM, Ekelund U, Harrison F, Harvey I, Panter J, Wareham NJ: **Physical activity and dietary behaviour in a population-based sample of British 10-year old children: The SPEEDY study (Sport, Physical activity and Eating behaviour: environmental Determinants in Young people)**. *BMC Public Health.* 2008, **8**(1):388.

46. The EuroQol Group: **EuroQol - a new facility for the measurement of health-related quality of life**. *Health Policy.* 1990, **16**(3):199-208.

47. Brooks R, The EuroQol Group: **EuroQol: The current state of play**. *Health Policy.* 1996, **37**(1):53-72.

48. Stevens KJ: **Working with children to develop dimensions for a preference-based, generic, pediatric, health-related quality-of-life measure**. *Qual Health Res.* 2010, **20**(3):340-351.

49. Stevens KJ: **Assessing the performance of a new generic measure of health-related quality of life for children and refining it for use in health state valuation**. *Appl Health Econ Health Pol.* 2011, **9**(3):157-169.

For peer review only

For peer review only - http://bmjopen.bmj.com/site/about/guidelines.xhtml

50. Corder K, van Sluijs EM, McMinn AM, Ekelund U, Cassidy A, Griffin SJ: **Perception versus reality: Awareness of physical activity levels of British children**. *Am J Prev Med.* 2010, **38**(1):1-8.

51. Godino JG, Watkinson C, Corder K, Sutton S, Griffin SJ, Van Sluijs EM: **Awareness of physical activity in healthy middle-aged adults: a cross-sectional study of associations with sociodemographic, biological, behavioural, and psychological factors**. *BMC Public Health* 2014, **14**:421.

52. D'Haese S, Gheysen F, De Bourdeaudhuij I, Deforche B, Van Dyck D, Cardon G: **The moderating effect of psychosocial factors in the relation between neighborhood walkability and children's physical activity**. *Int J Behav Nutr Phy.* 2016, **13**(1):128.

53. Motl RW, Dishman RK, Trost SG, Saunders RP, Dowda M, Felton G, Ward DS, Pate RR: **Factorial validity and invariance of questionnaires measuring social-cognitive determinants of physical activity among adolescent girls**. *Prev Med.* 2000, **31**(5):584-594.

54. Markland D, Tobin V: **A modification to the behavioural regulation in exercise questionnaire to include an assessment of amotivation**. *J Sport Exerc Psychol.* 2004, **26**(2):191-196.

55. Sebire SJ, Jago R, Fox KR, Edwards MJ, Thompson JL: **Testing a self-determination theory model of children's physical activity motivation: A cross-sectional study**. *Int J Behav Nutr Phy.* 2013, **10**:111.

56. Grotevant HD, Cooper CR: **Patterns of interaction in family relationships and the development of identity exploration in adolescence**. *Child Dev.* 1985, **56**(2):415-428.

57. Bengston PL, Grotevant HD: **The Individuality and Connectedness Q-sort: A measure for assessing individuality and connectedness in dyadic relationships**. *Pers Relationship.* 1999, **6**(2):213-225.

58. Beech BM, Klesges RC, Kumanyika SK, Murray DM, Klesges L, McClanahan B, Slawson D, Nunnally C, Rochon J, McLain-Allen B: **Child-and parent-targeted interventions: The Memphis GEMS pilot study**. *Ethnic Dis.* 2003, **13**(1; SUPP/1):S1-40.

For peer review only

For peer review only - http://bmjopen.bmj.com/site/about/guidelines.xhtml

59. Rhodes RE, Naylor P-J, McKay HA: **Pilot study of a family physical activity planning intervention among parents and their children**. *J Behav Med.* 2010, **33**(2):91-100.

60. Wilson EC: **A practical guide to value of information analysis**. *PharmacoEconomics* 2015, **33**(2):105-121.

61. Côté J, Salmela JH, Baria A, Russell S: **Organizing and interpreting unstructured qualitative data**. *Sport Psychol.* 1993, **7**:127-137.

62. National Institute for Health and Care Excellence: **Promoting physical activity for children and young people: Evidence update.** 2015. https://www.nice.org.uk/guidance/ph17/evidence/ph17-promoting-physical-activity-forchildren-and-young-people-evidence-update2. ccessed 8 Jan 2019.

For peer review only

Table 1.

Summary of FRESH Pilot Trial Intervention Components[1].

Intervention Components	Dose	Description	Behaviour Change Techniques	Targeted SDT constructs	Hypothesised mediators
1. 'Family time'	Minimum 1x/week, 10-20 mins	'Family time' is expected to provide a weekly (at minimum) opportunity for index children[2] and family members to review, revise, and update their family action planners. Family action planners prompt families to plan PA, monitor weekly steps, discuss any potential upcoming PA barriers and strategies to overcome them. Index children will be allocated as their family's 'team captain(s)' leading in challenge selection and uploading steps on the FRESH website.	Goal-setting<br><br>Self-monitoring<br><br>Positive feedback on progress<br><br>Social support<br><br>Praise<br><br>Positive reinforcement	Perceived competence<br><br>Perceived relatedness<br><br>Perceived autonomy	Social support<br><br>Family social norms for PA<br><br>PA awareness<br><br>Basic needs satisfaction<br><br>PA motivation
2. FRESH website	Minimum 1x/week, 5-20 mins	The FRESH website will provide a place for families to self-monitor their step counts and set goals by selecting challenges of varying difficulty. Specifically, the website allows families to 'walk' around the world by choosing one of three target cities to 'walk to' weekly. The challenges are framed as an easy, moderate, or hard challenge, which represents a 0%, 5%, or 10% increase, respectively, relative to the average steps they take in preceding weeks. Once adults and children accumulate an average of 10,000 and 12,000 steps/day, the step challenge increases will be reduced to 0%, 2.5%, and 5%, respectively. Families will also have access to:<br>• a general resources area with suggestions for activities that families could do together.<br>• a map for a visual representation showing the locations families have travelled to.<br>• a step calculator that will convert activities not captured by	Goal-setting<br><br>Self-monitoring<br><br>Positive feedback on progress<br><br>Rewards	Perceived competence<br><br>Perceived relatedness<br><br>Perceived autonomy	Social support<br><br>Family social norms for PA<br><br>PA awareness<br><br>Basic needs satisfaction<br><br>PA motivation

For peer review only - http://bmjopen.bmj.com/site/about/guidelines.xhtml

		pedometers to steps (e.g., swimming).<br>• their families' **step history** by challenge.<br>• a **leaderboard**. Families will opt in to being included in a leaderboard that is updated weekly, where families get points for: selecting a challenge, uploading steps, completing a challenge, accumulating a family average of 10,000 steps/day over the week, increasing steps from the previous week, and going on streaks (e.g., 5 completed challenges in a row).<br>Families with an ongoing challenge will receive **email reminders** to log steps three days and one day before an impending challenge ends.			
3. Pedometry	Throughout intervention (8 weeks)	All family members will receive pedometers to enable self-monitoring and provide immediate feedback. To allow families to view their progress towards their proximal and distal step goals, they will be encouraged to log their steps onto the FRESH website and/or onto the family action planners.	Self-monitoring<br><br>Immediate feedback	Perceived competence<br><br>Perceived autonomy	Social support<br><br>Family social norms for PA<br><br>PA awareness<br><br>Basic needs satisfaction<br><br>PA motivation
4. Competence reinforcement/ rewards	~1x/week (8 weeks)	After completing a challenge or if the challenge week ended, to praise effort (i.e., competence reinforcement), children received **personalised supportive letters in the mail** and messages on the FRESH website.<br>They also received small online and **tangible rewards**.<br>• Online rewards will include virtual passport stamps (i.e., virtual rewards) and access to reinforcement materials (i.e., interactive information about the cities they have walked past during their challenge).<br>• **Tangible rewards** will be in the form of collectable FRESH cards, which display the city names corresponding with the families' challenge. The cards will also enable family to play a card game version of 'rock, paper, scissors'.<br>Children will receive 2-4 passport stamps/cards for completed challenges (i.e., as difficulty increased, more stamps were awarded) and 1 passport stamp/card for an incomplete challenge.	Feedback on progress<br><br>Rewards	Perceived competence	Basic needs satisfaction<br><br>PA awareness

For peer review only

Note. [1]This table has been adapted from our feasibility study [21]; bolded text indicates new elements added for the FRESH pilot trial (i.e., post-feasibility study). [2]The index child refers to the child aged 7-11 years in the family. **Abbreviations.** PA, physical activity; SDT, Self-Determination Theory.

For peer review only

Table 2.

Order of Measures and Estimated Duration of Data Collection[1,2].

Measure	Duration
1. Anthropometric measures (height, weight, waist circumference)	5 mins/person
2. Questionnaire	20 mins/family
3. Blood pressure[3]	10 mins/person
4. Step Test (aerobic fitness)	Prep: 5 mins/person<br>Test: 10 mins/family
5. Accelerometer and GPS explanation	5 mins/family
6. Fictional Family Holiday (family functioning)	10 mins/family
**Total duration of baseline measurement session (includes consent/assent process)**	**120 mins**
**Total duration of subsequent measurement sessions**	**105 mins**

Note. [1]This table has been adapted from our feasibility study [21]. [2]Estimate based on a four-person household, total time increases by ~30 minutes per additional family member. [3]Duration is halved when calculating total duration because multiple monitors will be used to enable two family members to be measured simultaneously.

For peer review only

For peer review only - http://bmjopen.bmj.com/site/about/guidelines.xhtml

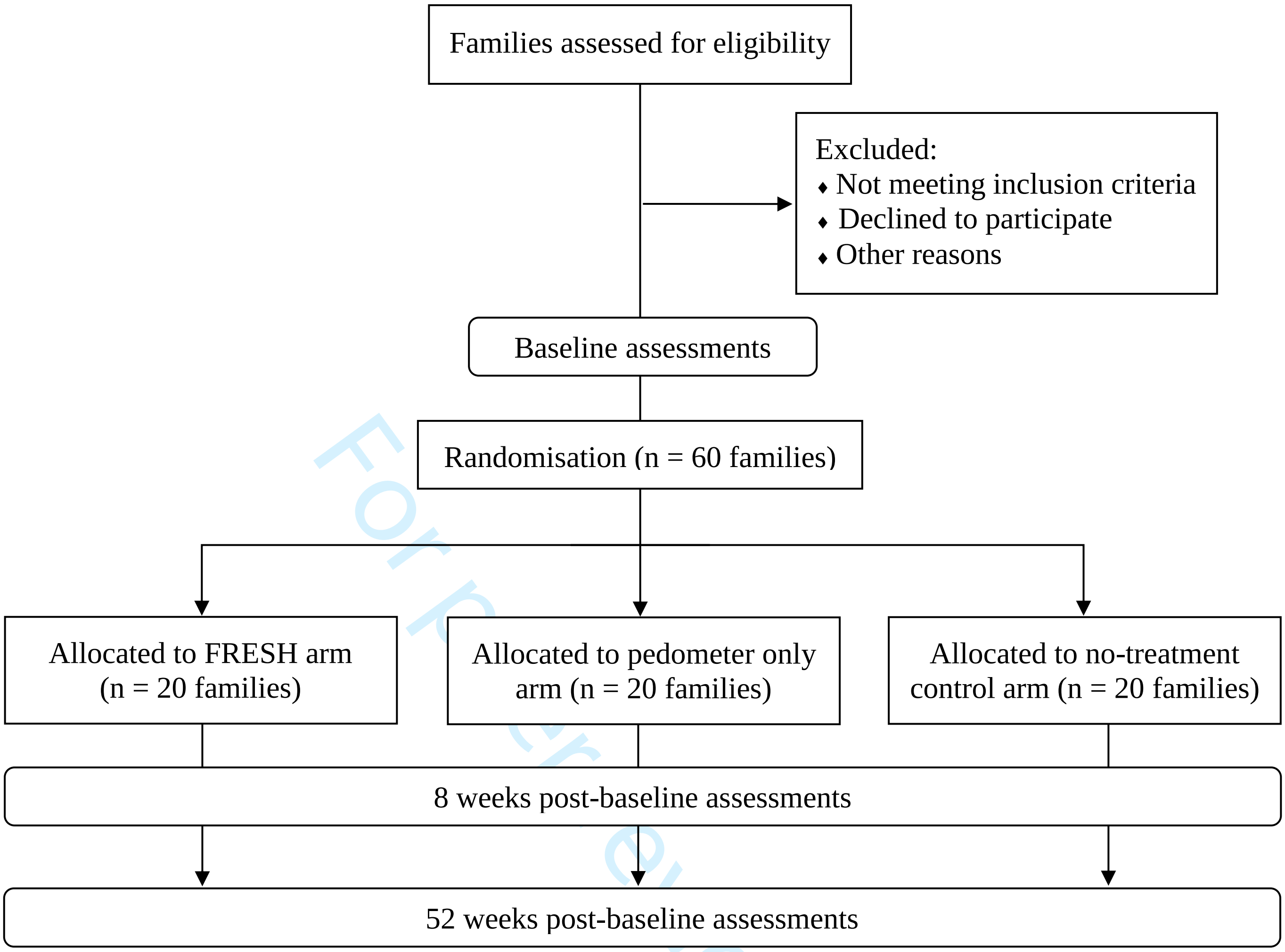

For peer review only

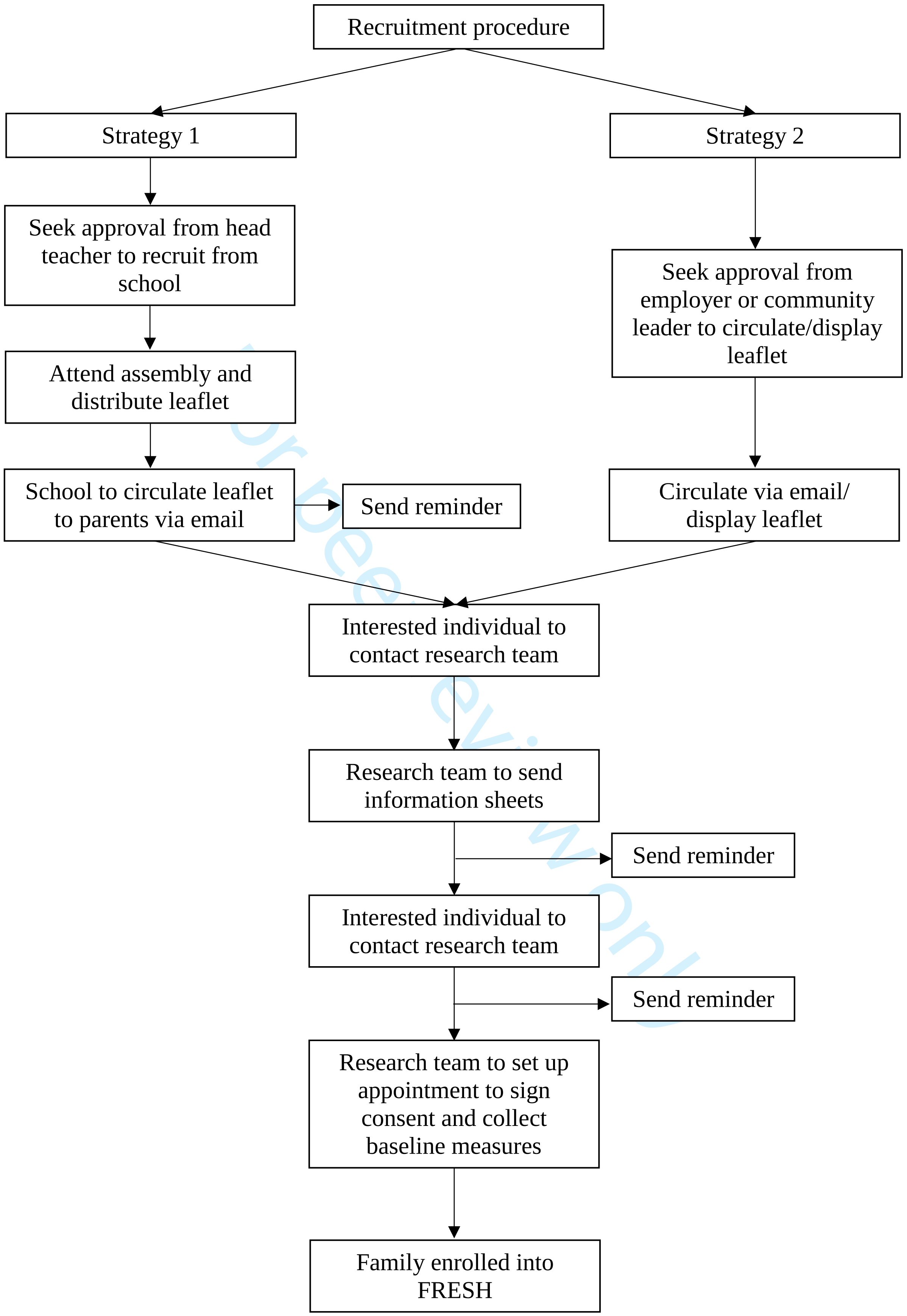
Recruitment procedure
Strategy 1
Strategy 2
Seek approval from head teacher to recruit from school
Attend assembly and distribute leaflet
School to circulate leaflet to parents via email
Send reminder
Seek approval from employer or community leader to circulate/display leaflet
Circulate via email/ display leaflet
Interested individual to contact research team
Research team to send information sheets
Send reminder
Interested individual to contact research team
Send reminder
Research team to set up appointment to sign consent and collect baseline measures
Family enrolled into FRESH

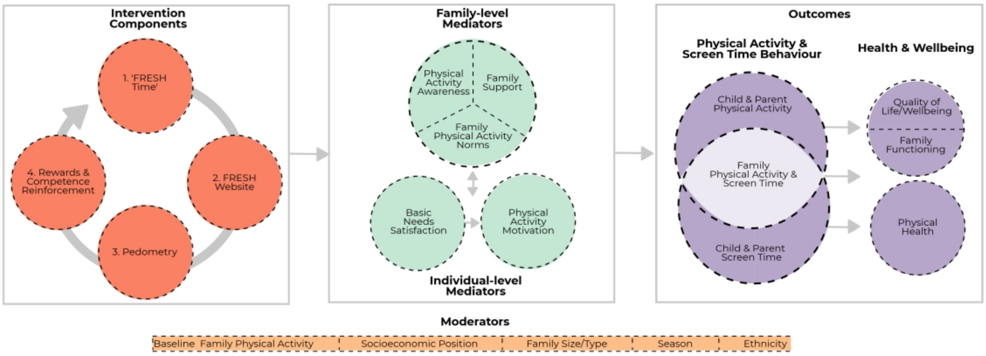

Figure 3. FRESH theoretical model.

1187x833mm (72 x 72 DPI)

Supplementary File – Parental and Young persons' questionnaires

For peer review only

___ ___ / ___ ___ / ___ ___ ___ ___
DD MM YYYY

TIME: 1 / 2 / 3 ID: FR __ __ __ / __ __ __ __ / __

Parent Questionnaire

- Please answer the questions as honestly and accurately as you can.
- All aspects of the study, including results, will be confidential and only the research team will have access to information on participants.
- Any reports or publications resulting from this study will not identify any individual who participated.
- Participation is entirely voluntary. There is no obligation to participate and withdrawal is permitted at any time without giving any reason and without any consequences.

For peer review only - http://bmjopen.bmj.com/site/about/guidelines.xhtml

Section 1: About you

1. In relation to the child that is between school Years 3-6 and participating in FRESH, this questionnaire is being completed by:

	Mother
	Father
	Step-mother
	Step-father
	Sister
	Brother
	Grandmother
	Grandfather
	Other (e.g., guardian). Please specify: ______________________________

2. What is your sex?

	Female
	Male
	Prefer not to say

3. When is your date of birth?

___ ___ / ___ ___ / ___ ___ ___ ___
DD MM YYYY

4. What is your ethnic origin? (Please tick **ONE** box only).

	White or White British
	Black or Black British
	Asian or Asian British
	Mixed
	Other ethnic group: _____________________________________
	Don't know
	Prefer not to answer

5. At what age did you finish full time education? __________ years.

	I have not finished full time education yet.

6. What best describes your current marital status? (Please tick the box which is most applicable).

	Single		Separated
	Married or living as married		Divorced
	Widowed		

For peer review only - http://bmjopen.bmj.com/site/about/guidelines.xhtml
For peer review only

ID: FR __ __ __ / __ __ __ __ / __

7. Describing your health **TODAY**. Under each heading, please tick the **ONE** box that best describes your health **TODAY**.

A. **Mobility (walking about)**

☐	I have no problems walking about today.
☐	I have slight problems walking about today.
☐	I have moderate problems walking about today.
☐	I have severe problems walking about today.
☐	I am unable to walk about today.

B. Self-Care

☐	I have no problems washing or dressing myself today.
☐	I have slight problems washing or dressing myself today.
☐	I have moderate problems washing or dressing myself today.
☐	I have severe problems washing or dressing myself today.
☐	I am unable to wash or dress myself today.

C. Usual activities (for example, work, study, housework, family or leisure activities).

☐	I have no problems doing my usual activities today.
☐	I have slight problems doing my usual activities today.
☐	I have moderate problems doing my usual activities today.
☐	I have severe problems doing my usual activities today.
☐	I am unable to do usual activities today.

D. Pain/discomfort

☐	I have no pain or discomfort today.
☐	I have slight pain or discomfort today.
☐	I have moderate pain or discomfort today.
☐	I have severe pain or discomfort today.
☐	I have extreme pain or discomfort today.

e. Anxiety/Depression

☐	I am not anxious or depressed today.
☐	I am slightly anxious or depressed today.
☐	I am moderately anxious or depressed today.
☐	I am severely anxious or depressed today.
☐	I am extremely anxious or depressed today.

For peer review only - http://bmjopen.bmj.com/site/about/guidelines.xhtml

f. We would like to know how good or bad your health is **TODAY**. This scale is numbered from 0 to 100. Mark an X on the scale to indicate how your health is **TODAY**.

100 means the **best** health you can imagine.
0 means the **worst** health you can imagine.

Now, please write the number you marked on the scale in the box below.

The best health you can imagine

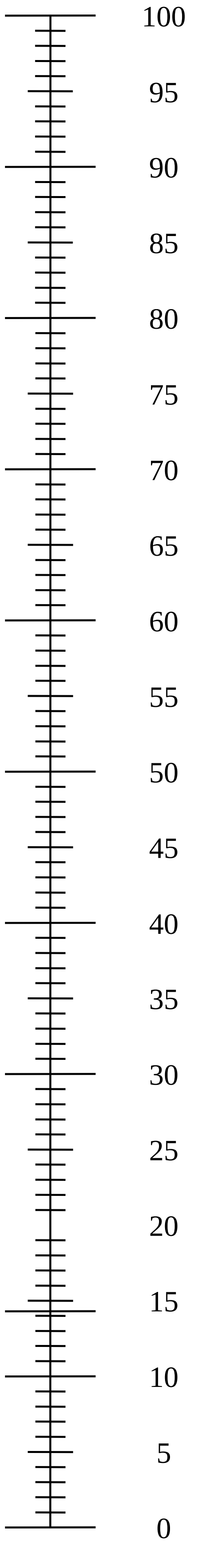

The worst health you can imagine

YOUR HEALTH TODAY = [ ]

For peer review only

For peer review only - http://bmjopen.bmj.com/site/about/guidelines.xhtml

ID: FR __ __ __ / __ __ __ __ / __

8. According to national recommendations adults should be active at a **moderate intensity** (e.g., cycling or fast walking) for **at least 150 minutes every week**.

Please indicate whether you think you have achieved this level of physical activity **over the last month**:

	**Yes**
	**No**

9. Why do you engage in physical activity? Please indicate how true or untrue these statements are to you. Please circle **ONE** number on each line.

	**Not true for me**		**A little true for me**		**Very true for me**
I feel guilty when I'm not physically active.	0	1	2	3	4
I value the benefits of physical activity.	0	1	2	3	4
I feel ashamed when I don't do the physical activity I planned to do.	0	1	2	3	4
It's important to me to be physically active regularly.	0	1	2	3	4
I can't see why I should bother being physically active.	0	1	2	3	4
I am physically active because others will not be pleased with me if I don't.	0	1	2	3	4
I don't see the point in being physically active.	0	1	2	3	4
I find physical activity to be enjoyable.	0	1	2	3	4
I feel under pressure from my friends/family to be physically active.	0	1	2	3	4
I feel content from participating in physical activity.	0	1	2	3	4

For peer review only - http://bmjopen.bmj.com/site/about/guidelines.xhtml

10. On average **over the last 4 weeks**, how much time did you spend on: (a) TV/video viewing and (b) computer use?

a. TV viewing or video watching. Please tick **ONE** box on each line.

**Hours of TV, DVD, or video watched (not on computer/ iPad, etc.) per day**	**Average over the last 4 weeks**					
	**None**	**Less than 1 hour per day**	**1 to 2 hours per day**	**2 to 3 hours per day**	**3 to 4 hours per day**	**More than 4 hours per day**
On a weekday before 6 pm						
On a weekday after 6 pm						
On a weekend before 6 pm						
On a weekend after 6 pm						

b. Computer use at home, but **not at work**. Please tick **ONE** box on each line. This includes use of: desktop computers or laptops, iPads, PlayStation, Xbox, Nintendo Wii or DS.

**Hours of home computer use or video watched (on computer/ iPad, etc.) per day**	**Average over the last 4 weeks**					
	**None**	**Less than 1 hour per day**	**1 to 2 hours per day**	**2 to 3 hours per day**	**3 to 4 hours per day**	**More than 4 hours per day**
On a weekday before 6 pm						
On a weekday after 6 pm						
On a weekend before 6 pm						
On a weekend after 6 pm						

For peer review only - http://bmjopen.bmj.com/site/about/guidelines.xhtml

ID: FR __ __ __ / __ __ __ __ / __

Section 2: About your child

In this section, '*your child*' is referring to your child in school Years 3-6 taking part in FRESH.

11. How physically active would you say **your child** is? Please tick one.

☐	Very inactive.
☐	Fairly inactive.
☐	Neither inactive or active.
☐	Fairly active.
☐	Very active.

12. Which of the following activities did **your child** do in the past 7 days? Please put '0' if not applicable.

Did **your child** do the following activities in the past 7 days?		**Monday-Friday** **Total hours/minutes**	**Saturday-Sunday** **Total hours/minutes**
**Example:** Watching TV/videos	**No** **Yes** (circled)	**15 hrs**	**6 hrs 30 mins**
Playing video games (e.g. PlayStation/Xbox/Nintendo DS).	No Yes		
Using computer/tablet.	No Yes		
Watching TV/DVD/video.	No Yes		
Playing games on the phone.	No Yes		
Other (please state): ______________________________.	No Yes		

13. Please circle **ONE** response indicating to how much you agree or disagree with each of these statements.

	**Strongly disagree**			**Strongly agree**
I limit how long my child plays video games (including PlayStation, Xbox, and Gameboy).	1	2	3	4
I limit how long my child can use the computer for things other than homework (such as playing computer games and surfing the internet).	1	2	3	4
I limit how long my child can watch TV or DVDs each day (including educational and non-educational programs).	1	2	3	4

For peer review only - http://bmjopen.bmj.com/site/about/guidelines.xhtml

Section 3: About your family

14. In an **average week**, how often do you do these activities **together as a family** (i.e., joint activities including at least one child and one other family member)? Please tick **ONE** box on each line.

	Number of Times each week		
	**0 times each week**	**1-3 times each week**	**4 or more times each week**
Play active games (like playing sports or tag).			
Go to the park.			
Play board games or cards.			
Go for a bike ride.			
Go for a walk or hike.			
Watch TV or movies.			
Have family talks.			
Go swimming.			
Walk the pet(s).			
Visit family or friends.			
Eat meals together.			
Walk or bike to school.			
Play computer or video games (like PlayStation/Xbox/Nintendo DS).			
**Other** (please state): ___________________________.			
**Other** (please state): ___________________________.			
**Other** (please state): ___________________________.			

15. I think my family should engage in physical activity regularly (on most or all days of the week) during their free time. Please circle **ONE**.

	Strongly disagree.
	Somewhat disagree.
	Neither agree or disagree.
	Somewhat agree.
	Strongly agree.

For peer review only - http://bmjopen.bmj.com/site/about/guidelines.xhtml

ID: FR __ __ __ / __ __ __ __ / __

16. Please circle **ONE** number to indicate how frequently or infrequently you do the following.

	**Never**	**Rarely**	**Sometimes**	**Often**	**Very often**
**How often…**					
…do you encourage someone in your family to be physically active (e.g., bike riding walking, playing sports)?	1	2	3	4	5
…are you physically active together with someone in your family?	1	2	3	4	5
…do you take someone in your family to places where they can be physically active (e.g., the park, sports practice, swimming lessons)?	1	2	3	4	5
…do you watch or cheer for someone in your family while they are doing physical activity (e.g., sports games, gymnastics/ dance competitions)?	1	2	3	4	5
…do you ask someone in your family to be physically active with you?	1	2	3	4	5
…do you tell someone in your family that they are doing well when they are doing physical activities?	1	2	3	4	5

For peer review only

For peer review only - http://bmjopen.bmj.com/site/about/guidelines.xhtml

MRC Epidemiology Unit UNIVERSITY OF CAMBRIDGE CEDAR Centre for Diet and Activity Research UEA University of East Anglia NHS National Institute for Health Research

___ ___ / ___ ___ / ___ ___ ___ ___
DD MM YYYY

TIME: 1 / 2 / 3 ID: FR __ __ __ / __ __ __ __ / __

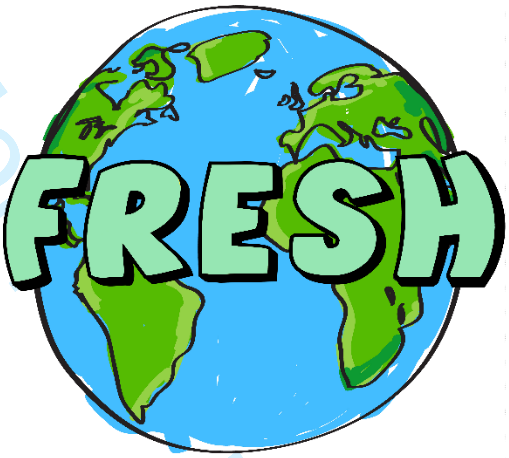

Young Persons Questionnaire
(15 years and under)

- Please answer the questions as honestly and accurately as you can.
- This is not a test, there is no right or wrong answer to the questions.
- If we're worried about any of the answers you have given about how you feel, we'll talk to you about it and may need to share with another adult.
- If the question asks about your parents, we mean your mum or dad or other adults who live and take care of you.

For peer review only

For peer review only - http://bmjopen.bmj.com/site/about/guidelines.xhtml

Physical Activity is any activity that increases your breathing and makes your heart beat faster.

Physical Activity can be done in sports, playing with friends, or walking to school.

Some examples of physical activity are running, walking quickly, cycling, skateboarding, dancing, swimming, basketball, football, gymnastics, and playing on the playground.

For peer review only - http://bmjopen.bmj.com/site/about/guidelines.xhtml

ID: FR __ __ __ / __ __ __ __ / __

Part 1: About you

1. When is your birthday? (For example 1st July) ___________________________

2. How old are you? ______________________ years old.

3. Are you a boy or a girl?

☐	Girl
☐	Boy
☐	Prefer not to say

4. The next questions ask about how you are **TODAY**. For each question, read all the choices and tick the box ☑ that is most like you today. Only tick **ONE** box for each question.

Example: Today I feel quite upset so I will tick this box.

Upset

☐	I don't feel upset today.
☐	I feel a little bit upset today.
☐	I feel a bit upset today.
✓	I feel quite upset today.
☐	I feel very upset today.

Now tick the **ONE** box that is most like you today.

A. How **worried** are you **TODAY**?

☐	I don't feel worried today.
☐	I feel a little bit worried today.
☐	I feel a bit worried today.
☐	I feel quite worried today.
☐	I feel very worried today.

B. How **sad** are you **TODAY?**

☐	I don't feel sad today.
☐	I feel a little bit sad today.
☐	I feel a bit sad today.
☐	I feel quite sad today.
☐	I feel very sad today.

C. How much **pain** are you in **TODAY?**

☐	I don't have any pain today.
☐	I have a little bit of pain today.
☐	I have a bit of pain today.
☐	I have quite a lot of pain today.
☐	I have a lot of pain today.

D. How **tired** are you **TODAY?**

For peer review only - http://bmjopen.bmj.com/site/about/guidelines.xhtml

- [ ] I don't feel tired today.
- [ ] I feel a little bit tired today.
- [ ] I feel a bit tired today.
- [ ] I feel quite tired today.
- [ ] I feel very tired today.

E. How **annoyed** are you **TODAY?**

- [ ] I don't feel annoyed today.
- [ ] I feel a little bit annoyed today.
- [ ] I feel a bit annoyed today.
- [ ] I feel quite annoyed today.
- [ ] I feel very annoyed today.

F. Did you have problems with your **school work/homework** (such as reading, writing, doing lessons) **TODAY?**

- [ ] I have no problems with my schoolwork/homework today.
- [ ] I have a few problems with my schoolwork/homework today.
- [ ] I have some problems with my schoolwork/homework today.
- [ ] I have many problems with my schoolwork/homework today.
- [ ] I can't do my schoolwork/homework today.

G. Did you have problems **sleeping LAST NIGHT?**

- [ ] Last night I had no problems sleeping.
- [ ] Last night I had a few problems sleeping.
- [ ] Last night I had some problems sleeping.
- [ ] Last night I had many problems sleeping.
- [ ] Last night I couldn't sleep at all.

H. Did you have problems with your **daily routine** (things like eating, having a bath/shower, getting dressed) **TODAY?**

- [ ] I have no problems with my daily routine today.
- [ ] I have a few problems with my daily routine today.
- [ ] I have some problems with my daily routine today.
- [ ] I have many problems with my daily routine today.
- [ ] I can't do my daily routine today.

I. How able are you to **join in activities** (things like playing with your friends, doing sports, joining in things) **TODAY**?

- [ ] I can join in with any activities today.
- [ ] I can join in with most activities today.
- [ ] I can join in with some activities today.
- [ ] I can join in with a few activities today.
- [ ] I can join in with no activities today.

For peer review only - http://bmjopen.bmj.com/site/about/guidelines.xhtml

ID: FR __ __ __ / __ __ __ __ / __

Part 2: You thoughts about physical activity

REMEMBER…

Physical Activity is any activity that increases your breathing and makes your heart beat faster.

Some examples of physical activity are running, brisk walking, cycling, skateboarding, dancing, swimming, basketball, football, gymnastics, and playing on the playground.

5. Compared with other boys or girls your age, would you say that you are:

	Much more active.
	More active.
	About average.
	Less active.
	Much less active.

6. How true is each sentence for you? Please circle **ONE** number each line.

	**Not true**	**A little bit true**	**Very true**
I am active because I enjoy being active.	1	2	3
I am active because it is important to me to be active.	1	2	3
I am active because when I don't do activity I feel bad about myself.	1	2	3
I am active because if I'm not, other people will not be happy with me.	1	2	3

7. How true is each sentence for you? Please circle **ONE** number each line.

	**Not true**	**A little bit true**	**Very true**
I can decide which activities I want to do.	1	2	3
I feel I am active because I want to be.	1	2	3
When it comes to being active, I think I am pretty good.	1	2	3
I am happy with how good I am at being active.	1	2	3
When it comes to being active, I feel like I'm part of a group.	1	2	3
When it comes to being active, I fit in well with others.	1	2	3

For peer review only - http://bmjopen.bmj.com/site/about/guidelines.xhtml

Part 3: About your parents/family

8. How often do **your parents** let you do the following? Please circle **ONE** number each line.

	Never	Sometimes	Often
My parents let me watch TV as much as I want.	1	2	3
My parents let me use the computer as much as I want for things that aren’t homework like playing games or watching videos.	1	2	3
My parents let me play video games (such as PlayStation, Xbox, and Gameboy) as much as I want.	1	2	3

9. How often does **your family** do the following? Please circle **ONE** number each line.

	Never	Sometimes	Often
How often does someone in your family encourage (or tell) you to do active things (like bike riding walking, playing sports)?	1	2	3
How often are you active together with someone in your family?	1	2	3
How often does someone in your family bring you to places where you can be active (like the park, sports practice, swimming lessons)?	1	2	3
How often does someone in your family watch or cheer for you when you are doing active things (like sports games or gymnastics or dance competitions)?	1	2	3
How often does someone in your family ask you to be active with them?	1	2	3
How often does someone in your family tell you that you are doing good when you are doing active things?	1	2	3

For peer review only - http://bmjopen.bmj.com/site/about/guidelines.xhtml

ID: FR __ __ __ / __ __ __ __ / __

10. In a normal week, how many times do you do these activities together as a family? Please tick **ONE** box every line.

	**Number of Times each week**		
	**0 times each week**	**1-3 times each week**	**4 or more times each week**
Play active games (like playing sports or tag).			
Go to the park.			
Play board games or cards.			
Go for a bike ride.			
Go for a walk or hike.			
Watch TV or movies.			
Have family talks.			
Go swimming.			
Walk the pet(s).			
Visit family or friends.			
Eat meals together.			
Walk or bike to school.			
Play computer or video games (like PlayStation/Xbox/Nintendo DS).			
**Other:** ___________________________.			
**Other:** ___________________________.			

11. How true is each sentence? Please fill in **ONE** circle in each line.

	**Not true**	**A little bit true**	**Very true**	**Does not apply**
My Mum thinks that I should be active during my free time on most days of the week.	1	2	3	
My Dad thinks that I should be active during my free time on most days of the week.	1	2	3	
My sister(s) think that I should be active during my free time on most days of the week.	1	2	3	
My brother(s) think that I should be active during my free time on most days of the week.	1	2	3	

For peer review only - http://bmjopen.bmj.com/site/about/guidelines.xhtml

STANDARD PROTOCOL ITEMS: RECOMMENDATIONS FOR INTERVENTIONAL TRIALS

SPIRIT 2013 Checklist: Recommended items to address in a clinical trial protocol and related documents*

Section/item	Item No	Description	Reported on page No
**Administrative information**			
Title	1	Descriptive title identifying the study design, population, interventions, and, if applicable, trial acronym	1
Trial registration	2a	Trial identifier and registry name. If not yet registered, name of intended registry	2,14
	2b	All items from the World Health Organization Trial Registration Data Set	N/A
Protocol version	3	Date and version identifier	N/A
Funding	4	Sources and types of financial, material, and other support	15
Roles and responsibilities	5a	Names, affiliations, and roles of protocol contributors	1
	5b	Name and contact information for the trial sponsor	15
	5c	Role of study sponsor and funders, if any, in study design; collection, management, analysis, and interpretation of data; writing of the report; and the decision to submit the report for publication, including whether they will have ultimate authority over any of these activities	15
	5d	Composition, roles, and responsibilities of the coordinating centre, steering committee, endpoint adjudication committee, data management team, and other individuals or groups overseeing the trial, if applicable (see Item 21a for data monitoring committee)	N/A
**Introduction**			
Background and rationale	6a	Description of research question and justification for undertaking the trial, including summary of relevant studies (published and unpublished) examining benefits and harms for each intervention	4
	6b	Explanation for choice of comparators	N/A
Objectives	7	Specific objectives or hypotheses	4

For peer review only

Trial design	8	Description of trial design including type of trial (eg, parallel group, crossover, factorial, single group), allocation ratio, and framework (eg, superiority, equivalence, noninferiority, exploratory)	5
**Methods: Participants, interventions, and outcomes**			
Study setting	9	Description of study settings (eg, community clinic, academic hospital) and list of countries where data will be collected. Reference to where list of study sites can be obtained	5-6
Eligibility criteria	10	Inclusion and exclusion criteria for participants. If applicable, eligibility criteria for study centres and individuals who will perform the interventions (eg, surgeons, psychotherapists)	5
Interventions	11a	Interventions for each group with sufficient detail to allow replication, including how and when they will be administered	7-8, Table 1, Figure 3
	11b	Criteria for discontinuing or modifying allocated interventions for a given trial participant (eg, drug dose change in response to harms, participant request, or improving/worsening disease)	N/A
	11c	Strategies to improve adherence to intervention protocols, and any procedures for monitoring adherence (eg, drug tablet return, laboratory tests)	7-8, Table 1
	11d	Relevant concomitant care and interventions that are permitted or prohibited during the trial	
Outcomes	12	Primary, secondary, and other outcomes, including the specific measurement variable (eg, systolic blood pressure), analysis metric (eg, change from baseline, final value, time to event), method of aggregation (eg, median, proportion), and time point for each outcome. Explanation of the clinical relevance of chosen efficacy and harm outcomes is strongly recommended	9-11, Table 2
Participant timeline	13	Time schedule of enrolment, interventions (including any run-ins and washouts), assessments, and visits for participants. A schematic diagram is highly recommended (see Figure)	Figure 1
Sample size	14	Estimated number of participants needed to achieve study objectives and how it was determined, including clinical and statistical assumptions supporting any sample size calculations	12
Recruitment	15	Strategies for achieving adequate participant enrolment to reach target sample size	6-7, Figure 2
**Methods: Assignment of interventions (for controlled trials)**			
Allocation:			

Sequence generation	16a	Method of generating the allocation sequence (eg, computer-generated random numbers), and list of any factors for stratification. To reduce predictability of a random sequence, details of any planned restriction (eg, blocking) should be provided in a separate document that is unavailable to those who enrol participants or assign interventions	7
Allocation concealment mechanism	16b	Mechanism of implementing the allocation sequence (eg, central telephone; sequentially numbered, opaque, sealed envelopes), describing any steps to conceal the sequence until interventions are assigned	7
Implementation	16c	Who will generate the allocation sequence, who will enrol participants, and who will assign participants to interventions	7
Blinding (masking)	17a	Who will be blinded after assignment to interventions (eg, trial participants, care providers, outcome assessors, data analysts), and how	12
	17b	If blinded, circumstances under which unblinding is permissible, and procedure for revealing a participant's allocated intervention during the trial	12
**Methods: Data collection, management, and analysis**			
Data collection methods	18a	Plans for assessment and collection of outcome, baseline, and other trial data, including any related processes to promote data quality (eg, duplicate measurements, training of assessors) and a description of study instruments (eg, questionnaires, laboratory tests) along with their reliability and validity, if known. Reference to where data collection forms can be found, if not in the protocol	9-11, Table 2
	18b	Plans to promote participant retention and complete follow-up, including list of any outcome data to be collected for participants who discontinue or deviate from intervention protocols	7
Data management	19	Plans for data entry, coding, security, and storage, including any related processes to promote data quality (eg, double data entry; range checks for data values). Reference to where details of data management procedures can be found, if not in the protocol	N/A
Statistical methods	20a	Statistical methods for analysing primary and secondary outcomes. Reference to where other details of the statistical analysis plan can be found, if not in the protocol	12-13
	20b	Methods for any additional analyses (eg, subgroup and adjusted analyses)	N/A
	20c	Definition of analysis population relating to protocol non-adherence (eg, as randomised analysis), and any statistical methods to handle missing data (eg, multiple imputation)	N/A

For peer review only - http://bmjopen.bmj.com/site/about/guidelines.xhtml

Methods: Monitoring

Data monitoring	21a	Composition of data monitoring committee (DMC); summary of its role and reporting structure; statement of whether it is independent from the sponsor and competing interests; and reference to where further details about its charter can be found, if not in the protocol. Alternatively, an explanation of why a DMC is not needed	N/A
	21b	Description of any interim analyses and stopping guidelines, including who will have access to these interim results and make the final decision to terminate the trial	N/A
Harms	22	Plans for collecting, assessing, reporting, and managing solicited and spontaneously reported adverse events and other unintended effects of trial interventions or trial conduct	N/A
Auditing	23	Frequency and procedures for auditing trial conduct, if any, and whether the process will be independent from investigators and the sponsor	N/A

Ethics and dissemination

Research ethics approval	24	Plans for seeking research ethics committee/institutional review board (REC/IRB) approval	2, 14
Protocol amendments	25	Plans for communicating important protocol modifications (eg, changes to eligibility criteria, outcomes, analyses) to relevant parties (eg, investigators, REC/IRBs, trial participants, trial registries, journals, regulators)	N/A
Consent or assent	26a	Who will obtain informed consent or assent from potential trial participants or authorised surrogates, and how (see Item 32)	5, 7, 14
	26b	Additional consent provisions for collection and use of participant data and biological specimens in ancillary studies, if applicable	
Confidentiality	27	How personal information about potential and enrolled participants will be collected, shared, and maintained in order to protect confidentiality before, during, and after the trial	14
Declaration of interests	28	Financial and other competing interests for principal investigators for the overall trial and each study site	15
Access to data	29	Statement of who will have access to the final trial dataset, and disclosure of contractual agreements that limit such access for investigators	N/A
Ancillary and post-trial care	30	Provisions, if any, for ancillary and post-trial care, and for compensation to those who suffer harm from trial participation	N/A

For peer review only

Dissemination policy	31a	Plans for investigators and sponsor to communicate trial results to participants, healthcare professionals, the public, and other relevant groups (eg, via publication, reporting in results databases, or other data sharing arrangements), including any publication restrictions	14
	31b	Authorship eligibility guidelines and any intended use of professional writers	N/A
	31c	Plans, if any, for granting public access to the full protocol, participant-level dataset, and statistical code	N/A
**Appendices**			
Informed consent materials	32	Model consent form and other related documentation given to participants and authorised surrogates	N/A
Biological specimens	33	Plans for collection, laboratory evaluation, and storage of biological specimens for genetic or molecular analysis in the current trial and for future use in ancillary studies, if applicable	N/A

*It is strongly recommended that this checklist be read in conjunction with the SPIRIT 2013 Explanation & Elaboration for important clarification on the items. Amendments to the protocol should be tracked and dated. The SPIRIT checklist is copyrighted by the SPIRIT Group under the Creative Commons "Attribution-NonCommercial-NoDerivs 3.0 Unported" license.

For peer review only
